# Single-cell transcriptome profiling of the human developing spinal cord reveals a conserved genetic programme with human-specific features

**DOI:** 10.1242/dev.199711

**Published:** 2021-08-05

**Authors:** Teresa Rayon, Rory J. Maizels, Christopher Barrington, James Briscoe

**Affiliations:** The Francis Crick Institute, London NW1 1AT, UK

**Keywords:** Developmental patterning, Human, Neuronal subtype identity, Single-cell transcriptome, Spinal cord

## Abstract

The spinal cord receives input from peripheral sensory neurons and controls motor output by regulating muscle innervating motor neurons. These functions are carried out by neural circuits comprising molecularly distinct neuronal subtypes generated in a characteristic spatiotemporal arrangement from progenitors in the embryonic neural tube. To gain insight into the diversity and complexity of cells in the developing human neural tube, we used single-cell mRNA sequencing to profile cervical and thoracic regions in four human embryos of Carnegie stages (CS) CS12, CS14, CS17 and CS19 from gestational weeks 4-7. Analysis of progenitor and neuronal populations from the neural tube and dorsal root ganglia identified dozens of distinct cell types and facilitated the reconstruction of the differentiation pathways of specific neuronal subtypes. Comparison with mouse revealed overall similarity of mammalian neural tube development while highlighting some human-specific features. These data provide a catalogue of gene expression and cell type identity in the human neural tube that will support future studies of sensory and motor control systems. The data can be explored at https://shiny.crick.ac.uk/scviewer/neuraltube/.

## Abstract (precis)

**Summary:** Single-cell transcriptome analysis of the neural tube of human embryos from gestational weeks 4-7 provides insight into the molecular complexity and cellular diversity of the developing human spinal cord.

## INTRODUCTION

The spinal cord receives and processes information from sensory neurons in the peripheral nervous system (PNS) and controls muscle movement by coordinating the activity of motor neurons (MNs). The neural circuits that perform these tasks comprise molecularly and physiologically distinct classes of neurons that are generated in a stereotypic spatial and temporal order from proliferating progenitors in the embryonic neural tube and neural crest. The spatiotemporal arrangement of progenitors and the identity of the neurons they generate is determined by gene regulatory networks (GRNs) controlled by extrinsic signals (Delás and Briscoe, 2020; Sagner and Briscoe, 2019). The composition and operation of these GRNs have been studied in model organisms, such as mouse, chick and zebrafish, and recent work has included the systematic profiling of single cells from the mouse neural tube and neural crest (Delile et al., 2019; Ray et al., 2018; Rosenberg et al., 2018; Sharma et al., 2020; Soldatov et al., 2019). This has provided catalogues of gene expression, revealed complexity, and allowed the detailed molecular classification of multiple cell types.

Although recent attention has focused on profiling the transcriptomes of cells in several regions of the developing human brain (Eze et al., 2021), the embryonic human spinal cord and PNS have been less well described. The available molecular characterisation has shown that the identity and overall organisation of progenitors and neurons is, in the main, similar between humans and other vertebrates (Dady et al., 2021 preprint; Marklund et al., 2014; Rayon et al., 2020). Nevertheless, the extent of the similarity in the molecular composition of cells in mouse and human neural tubes has not been determined. Notably, several features of human neural tube development have been reported to differ from other species. For example, a subset of neural progenitors in the spinal cord co-express the transcription factors OLIG2 and NKX2-2 in human embryos. These are relatively rare at the equivalent developmental stages in the mouse and chick (Marklund et al., 2014).

Systematic profiling of cell identity in the developing nervous system of human embryos would offer a clearer picture of the developing human neural tube and allow comparisons with other species. To this end, we profiled gene expression in single-cell transcriptomes in samples of dissected trunks from human embryos from gestational weeks 4 to 7. We recovered a total of 71,219 cells, of which 43,485 were neural, from four embryos and used these to generate a single-cell atlas of the developing human spinal cord and dorsal root ganglia. Comparison with equivalent staged mouse embryos identified similarities and differences. To allow the data to be explored further, we developed an online resource that is available at https://shiny.crick.ac.uk/scviewer/neuraltube/.

## RESULTS AND DISCUSSION

### Identification of cell types in developing human embryos

To identify the cell types in the developing human spinal cord, we performed single-cell RNA sequencing (scRNA-seq) using the droplet-based 10x Chromium platform. We microdissected cervical-thoracic regions from shoulder to hip level of single embryos during the first trimester of human development, spanning 4 weeks of development from gestational weeks 4 to 7, corresponding to Carnegie stages (CS) 12, 14, 17 and 19. The samples from CS12, CS14 and CS19 were processed on the same day as the termination of pregnancy, whereas CS17 was processed for sequencing after a 24 h delay. We dissected CS14, CS17 and CS19 samples into anterior and posterior regions and sequenced these separately. Because the CS12 sample was smaller in size, to minimise cell loss we avoided further regional microdissection and sequenced cells of the entire trunk element. In addition, to compensate for the increased cell numbers of the older embryos, two replicates per time point were generated for the CS17 and the CS19 samples. In total, 120,620 droplets were annotated as cells after sequencing. To each sample, we applied similar quality filters to those we established for the developing mouse spinal cord (Delile et al., 2019). This resulted in a dataset of 71,219 cells: 11,963 from CS12; 11,525 from CS14; 28,800 from CS17; and 18,931 from CS19 (Fig. S1A-D). More cells were detected in the CS17 sample compared with the other samples. This was likely due to the increased ambient RNA as indicated by increased dispersion in the proportion of mitochondrial unique molecular identifiers (UMIs). The total number of detected genes (median ∼3000 per cell) and UMIs in cells were similar in all samples analysed (Fig. S1A,B), and were similar to the mouse single-cell dataset (Delile et al., 2019). Expression of the male-specific *SRY* gene (Berta et al., 1990; Gubbay et al., 1990; Sinclair et al., 1990) was detected in CS12 and CS17 embryos, indicating these samples were male, whereas the lack of *SRY* expression in the CS14 and CS19 samples indicated that these were female (Fig. S1F).

**Fig. 1.**
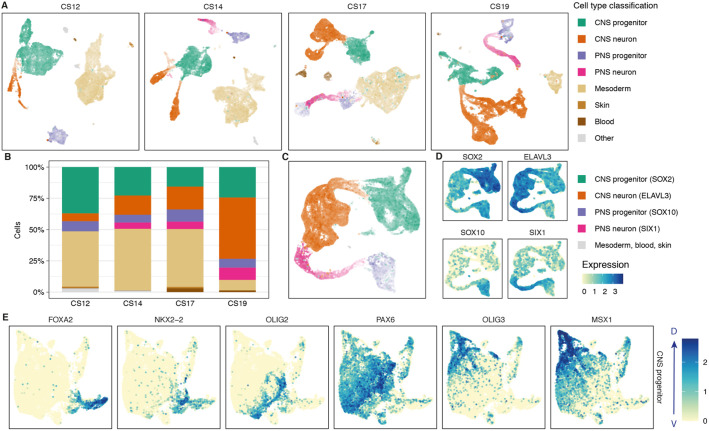
**Single-cell RNA-seq from the human developing spinal cord.** (A) UMAP of CS12, CS14, CS17 and CS19 samples annotated by gene module score. (B) Stacked bar chart indicating the cell type composition per time point (colour key as in A). (C) UMAP of the combined human neural dataset coloured by gene module score. Colour and brightness of cells reflects the representative gene module (*n*=43,485). (D) Feature plots of markers of neural progenitors (*SOX2*), neurons (*ELAVL3*) and neural crest progenitors (*SOX10*) and neurons (*SIX1*). (E) Feature plots of the spinal cord progenitor UMAP showing the patterned expression of the indicated DV patterning markers.

In a first step, we visualised each sample separately using uniform manifold approximation and projection (UMAP). To allocate cell types within this embedding, we calculated a gene module score (see Materials and Methods) that included the marker genes used to characterise tissue types in mouse (Delile et al., 2019). This identified progenitors and neurons of the spinal cord and dorsal root ganglion, mesoderm, haematopoietic and skin cells (Fig. 1A). We labelled as ‘other’ cells with poorly resolved transcriptomes and cells derived from other tissues of the embryo. Each of the samples displayed a similar composition of cell types (Fig. 1A) but with obvious differences in the proportions at different time points (Fig. 1B). More neurons were contained in the CS19 dataset, consistent with the progression of neurogenesis in the spinal cord over time. By contrast, mesodermal cells were enriched in the early stages (CS12 and CS14) compared with the late time points (Fig. 1B). This is due to the proximity of the mesoderm and neural tube at early stages, and the greater difficulty of cleanly dissecting smaller embryos.

To generate a spatiotemporal gene expression atlas of developing neural cells, we selected the neural cell clusters from each stage and combined these into a single dataset for further analysis. The combined human UMAP of the neural dataset contained a total of 43,485 cells and allowed us to distinguish progenitors and neurons of both the neural tube and dorsal root ganglia (Fig. 1C). Cells from different samples, whether of the same or different sex, did not form distinct clusters within the embedding, which suggested minimal batch effects after data processing (Fig. S1G). Expression of the progenitor marker *SOX2* and the neuronal marker *ELAVL3* allowed the identification of progenitors and neurons within the map. Likewise, the expression of the neural crest markers *SOX10* and *SIX1* allowed us to discriminate progenitors and neurons of neural crest origin (6747 cells) versus spinal cord (32,928 cells) (Fig. 1D). Within the UMAP, neurons of both the central and peripheral nervous system projected to adjacent clusters, pointing to a convergence in the gene expression programmes of central and peripheral neurons (Fig. 1C).

### Classification of central nervous cells

Computationally isolating spinal cord progenitor cells from all four time points arranged cells in the UMAP in a way that resembled the dorsoventral (DV) patterning of the developing neural tube (Fig. 1E). Nevertheless, attempts to use standard unsupervised methods to cluster progenitors were unsuccessful at classifying cells into the known DV domains. Similar approaches with neurons were also unsuccessful. This suggested that, similar to mouse, the complexity and combinatorial expression profile of genes in both progenitors and neurons preclude the use of unsupervised approaches.

**Fig. 2.**
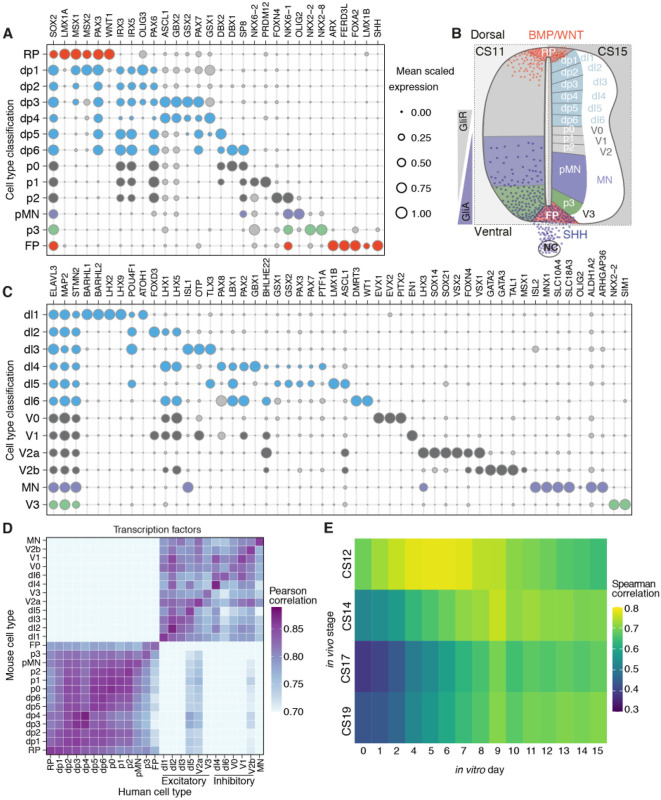
**Classification of dorsoventral progenitors and neuronal classes in human.** (A) Bubble plots indicating the expression of markers used to identify dorsoventral (DV) domains in human progenitors. (B) Diagram of the DV domains in the human developing spinal cord highlighting the opposing gradients of SHH and WNT/BMP and the patterning of the 11 DV domains, the floor plate (FP) and roof plate (RP). GliA, activator forms of Gli; GliR, Gli repressor forms; NC, notochord. (C) Bubble plots depicting the 11 neuronal classes in human. In A and C, genes chosen for cell assignment are coloured; grey circles correspond to markers not used for the selection of a specific population. Circle size indicates mean scaled gene expression levels. (D) Heatmap of the pairwise Pearson correlation coefficients of gene expression in mouse (vertical) and human (horizontal) progenitor and neural cells. (E) Heatmap of Spearman correlation coefficients per time point of the averaged gene expression of ventral progenitors and neurons in the human spinal cord *in vivo* (vertical) and *in vitro* (horizontal).

In order to classify subtype identity of neural cells, we adapted the ‘knowledge matrix’ we had previously constructed, using available molecular characterisation of the vertebrate developing neural tube (Delile et al., 2019) (Table S2). This represents an inventory in which each progenitor type and neuronal class is defined by the presence or absence of a characteristic set of marker genes (Fig. 2B). We binarised the expression levels of the marker gene set in the human transcriptome data and assigned each cell an identity from the knowledge matrix. This allowed the categorisation of cell types into specific progenitor and neuronal domains (Fig. 2A-C). Plotting the scaled mean expression of the genes used to assign cell identities revealed the expected conserved patterns of progenitor- and neuron-specific gene expression along the DV axis of the spinal cord (Fig. 2A-C). Overall, we observed similar identities and organisation of progenitors and neurons in mouse and human (Fig. S2A,B) with a mean scaled expression for most genes comparable between mouse and human. To compare the transcriptional similarity between progenitors and neurons of mouse and human, we used expression levels of homologous genes in each neural cell type in mouse and human and estimated the Pearson correlation coefficient for each pair of cell types. We measured the correlation using a set of transcription factors (see Materials and Methods). Mouse and human cells assigned to the same cell type had the highest correlation (Fig. 2D). The similarity of progenitor cells was higher between adjacent DV domains. By contrast, neuronal cell types clustered according to their excitatory or inhibitory function (Sagner and Briscoe, 2019) (Fig. 2D).

The availability of the human neural tube data allowed us to make comparisons with the differentiation of MNs *in vitro* from human embryonic stem cells. We selected highly expressed genes that varied during neurogenic differentiation from daily bulk RNA-seq samples of an *in vitro* differentiation of human MNs (Rayon et al., 2020). We then generated ‘pseudo bulk RNA’ samples from the human *in vivo* dataset by averaging gene expression of progenitors and neurons assigned to ventral domains (p3, pMN, p2, p1 and p0) at each time point. Pairwise comparison of *in vitro* and *in vivo* time points indicated that the CS12 sample had the highest correlation to *in vitro* Day 5 (Fig. 2E). CS14 was most similar to *in vitro* Day 9. Thus, the 4-day *in vitro* differentiation from Day 5 to Day 9 was similar to the developmental progression from CS12 to CS14, corresponding to gestational days 26-30 and days 31-35, respectively. By contrast, CS17 and CS19 stages, which correspond to gestational days 42-44 and day 48-51 of human development, showed higher correlations with later *in vitro* time points although there was less similarity at these stages. At least in part this could be because *in vitro* differentiations become progressively more asynchronous at later time points.

### Specific features of gene expression in the human spinal cord

**Fig. 3.**
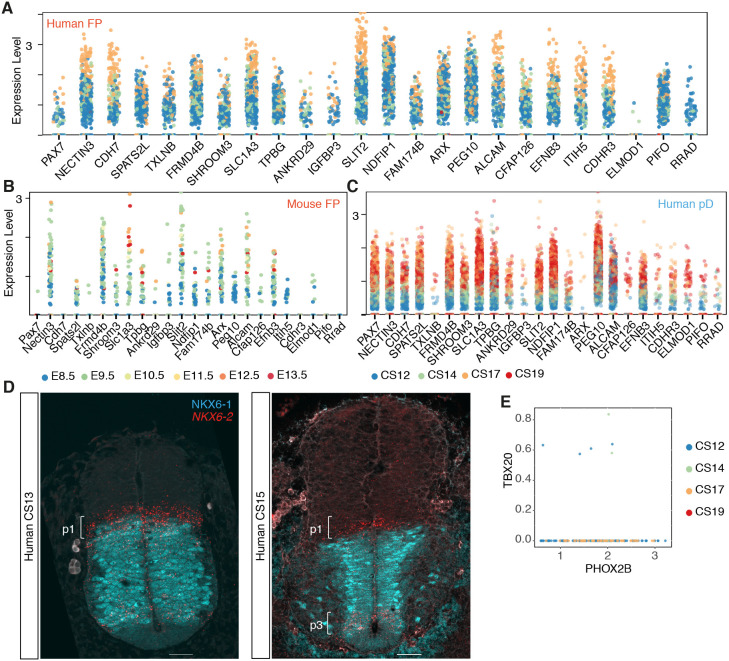
**Human-specific features of neural progenitors and visceral motor neurons.** (A-C) Genes most correlated with *PAX7* in human FP (A), mouse FP (B) and in human pD (dp1-dp6) (C). (D) NKX6-1 (cyan) and *NKX6*-2 (red) expression in transverse sections of the human neural tube at shoulder levels in CS13 (*n*=1, three sections) and CS15 (*n*=1, three sections) embryos. Scale bars: 50 µm. (E) Scatter plot of the expression of *PHOX2B* and *TBX20* in human transcriptomes (*n*=170 cells).

Although the transcriptomes of mouse and human neural cells were broadly similar, specific differences between mouse and human were apparent. *PAX7* expression was observed in dorsal progenitors of both mouse and human, but in addition *PAX7* expression was observed in floor plate (FP) cells of CS12, CS14 and CS17 human embryos. However, it was absent from mouse FP cells at all time points (Fig. 2A, Fig. S2A). This is consistent with immunohistochemical analysis of PAX7 in the human spinal cord from CS12 to CS15 (Betters et al., 2010; Dady et al., 2021 preprint). Inspection of genes correlating with *PAX7* in human FP cells highlighted several, including *CDH7*, *TXLNB*, *CDHR3*, *PIFO* and *RRAD*, that were expressed in human FP cells but not in mouse FP cells (Fig. 3A-C). Whether *PAX7* directly regulates these or other genes in human FP cells will require functional experiments.

*NKX6-2* expression in human was detected in p1, p2, pMN, p3 and FP progenitors (Fig. 2A). By contrast, mouse *Nkx6-2* defines p1 cells but is largely absent from other ventral progenitor domains (Fig. S2A,C) (Vallstedt et al., 2001). The expression of *NKX6-2* in the p1 domain of the human neural tube was maintained across the time points analysed but decreased over time in the pMN and p3 domains (Fig. 3D, Fig. S3B,C). The broad ventral expression profile of *NKX6-2* in the human neural progenitors resembled the broad expression of *Nkx6-2* in chick embryos and the transiently wider domain of expression of NKX6-2 at early development stages in the mouse (Vallstedt et al., 2001). This suggests that a heterochronic shift in the timing of *NKX6*-*2* downregulation could account for the difference in expression between mouse and human.

Other notable differences between human and mouse included the apparent scarce expression of *TBX20* in human visceral MNs. These MNs, present in the hindbrain and cervical spinal cord, are characterised by the expression of *Isl1*, *Phox2b*, *Tbx2*, *Tbx3* and *Tbx20* in mouse (Pontecorvi et al., 2008). Analysis of *PHOX2B*-expressing neurons in the human dataset revealed that although these cells expressed *TBX2* and *TBX3* there was little or no expression of *TBX20* (Fig. 3E, Fig. S3D).

### Expression of primate-specific genes

**Fig. 4.**
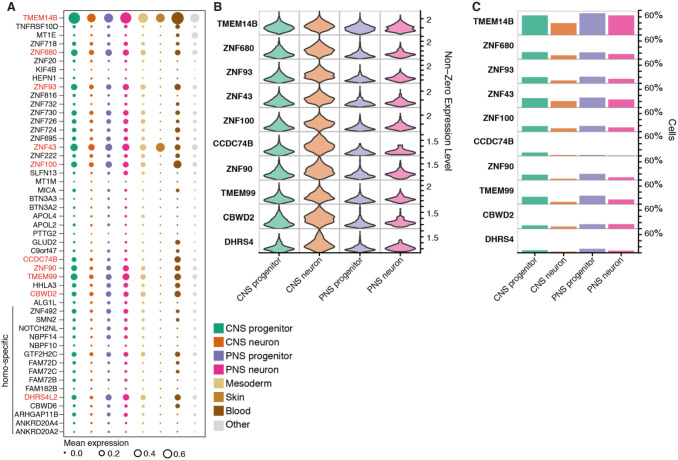
**Expression of primate-specific genes.** (A) Bubble plots indicating the expression of primate-specific genes in different cell types. Genes expressed at an average level of 0.10 transcripts/cell or higher in any cell type are highlighted in red. Circle size indicates mean scaled gene expression levels. (B) Violin plots for the expression of genes in neural cell types. Cells with null values for the genes were not included. (C) Proportion of cells within each cell type that express the indicated primate-specific genes.

The expression of genes specific to primates has received attention because of their potential role in species-specific aspects of human brain development, particularly in the neocortex (Fiddes et al., 2018; Florio et al., 2015; Heide et al., 2020; Liu et al., 2017; Suzuki et al., 2018). To investigate the expression of primate-specific genes in the more evolutionarily conserved spinal cord, we selected a list of 51 primate-specific protein-coding genes that have been shown to be enriched in human cerebral neural precursors (Florio et al., 2018; Liu et al., 2017) and assessed their expression. First, we examined the tissue specificity of expression. *TMEM133* (*ARHGAP42*) and *ZNF788* were not detectable in the dataset, suggesting they might be brain specific. Of the remaining 49 genes, 87% (43/49) were expressed broadly in more than one tissue (Fig. 4A), suggesting these were not specific to the nervous system. These included genes that have been shown to function in cortical expansion or folding in human (*TMEM14B*, *ARHGAP11B*, *NOTCH2NL*) (Fiddes et al., 2018; Florio et al., 2015; Heide et al., 2020; Liu et al., 2017; Suzuki et al., 2018). We examined more closely genes expressed at an average level of 0.10 transcripts/cell or greater in the central nervous system (CNS) or PNS (10 genes out of 49; 20%). The only gene expressed above this threshold with documented functions in brain development was *TMEM14B* (Fig. 4A). *TMEM14B*, along with the other nine genes, showed widespread expression in progenitors and neurons, with a higher percentage of progenitors expressing these genes (Fig. 4B,C). *CCDC74B*, *CBWD2*, *DHRS4* and *ZNF90* were somewhat more expressed in populations of CNS neurons compared with progenitors (Fig. 4B,C). Thus, expression of primate-specific genes implicated in developmental and neurogenic functions can be detected in the spinal cord and other tissues (Fig. S4A,B). The function of most of these genes remains unknown (Florio et al., 2018) and further analysis will be necessary.

### Classification of PNS cells

**Fig. 5.**
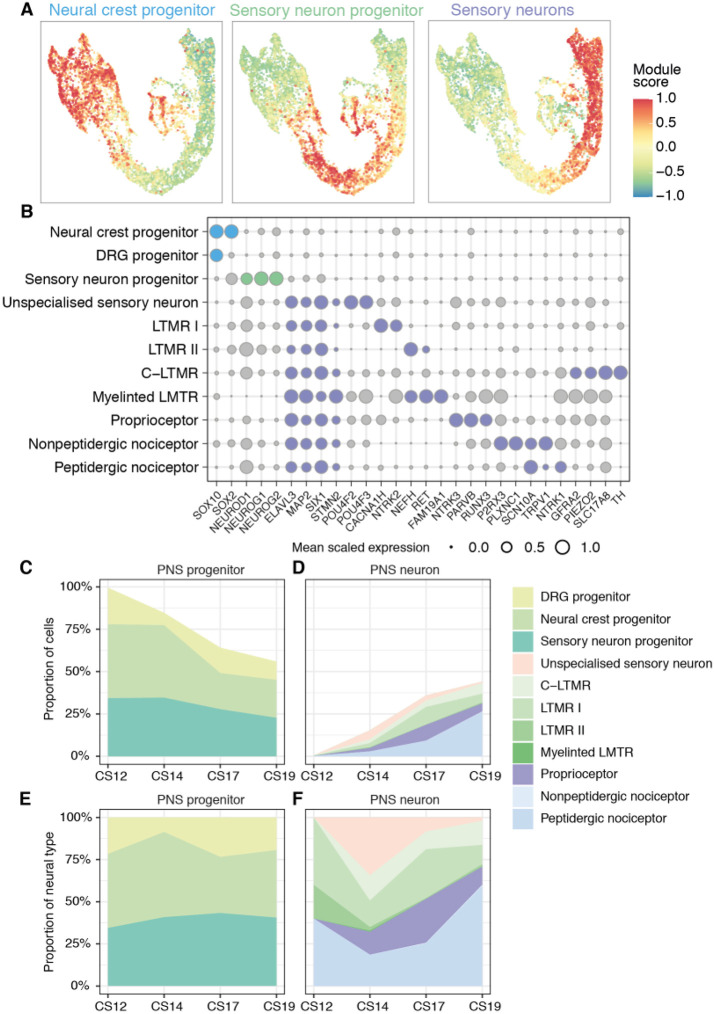
**Classification of peripheral nervous system cells in human.** (A) Gene module scores for progenitors, sensory neuron progenitors, and postmitotic sensory neurons in the peripheral nervous system (PNS) UMAP. (B) Bubble plots indicating the expression of markers used to identify PNS cell types. Genes chosen for cell assignment are coloured; grey circles correspond to markers not used for the selection of a specific population. Circle size indicates mean scaled gene expression levels. (C-F) Fractions of progenitors (C,E) and neurons (D,F) in the PNS during gestational weeks 4 to 7. For C and D, the data are proportional to the total neurons and progenitors at each time point. E and F show the same data as shown in C for progenitors or D for neurons as a proportion of the number of neural cells.

The PNS is derived from neural crest cells and emerges in trunk regions of human embryos around CS11-12 (O'Rahilly and Müller, 2007). We were therefore able to follow the differentiation dynamics of neural crest cells to PNS neurons during human embryogenesis. To classify the subtype identity of neural crest cells and investigate sensory neurogenesis in human, we mapped cells using markers of the PNS that had previously been defined in other species (Chiu et al., 2014; Hockley et al., 2019; Li et al., 2016; Usoskin et al., 2015; Vermeiren et al., 2020; Zeisel et al., 2018). We established a classification scheme to subdivide cells into the three cell types characteristic of the embryonic day (E) 11.5 mouse trunk dorsal root ganglia (Soldatov et al., 2019): (1) progenitors, marked by the expression of *SOX10* alone or in combination with *SOX2*; (2) sensory neuron precursors, marked by the expression of *NEUROG1*, *NEUROG2* and *NEUROD1*; and (3) postmitotic sensory neurons expressing *ELAVL3* (Fig. 5A), as the expression of the broad mouse somatosensory neuron marker advillin (AVIL) was limited to a small subset of cells in the human dataset (Fig. 5A).

We next established a knowledge matrix of dorsal root ganglia-expressed genes that distinguish cell types and used this to classify PNS cells into subtypes (Fig. 5B). Overall, the classification was similar to mouse, with several transcription factors characteristic of terminal neuronal subtypes in the adult expressed in embryonic sensory neurons (Fig. 5B, Fig. S5A). Gene expression programmes characteristic of mechanoreceptor (LTMRs), proprioceptor, peptidergic and non-peptidergic neurons were evident. Moreover, the expression profile of genes specific to distinct classes of PNS neurons agreed with recent findings of a single neurogenic trajectory from progenitors through a transcriptionally unspecialised state that then diversifies into distinct sensory lineages (Sharma et al., 2020; Soldatov et al., 2019).

The proportion of neural crest cells and neuronal progenitors decreased from CS12 to CS19 as the proportion of peripheral neurons increased (Fig. 5C-F). No PNS neurons were detected at the earliest time point (CS12), and a low percentage of terminal neuronal subtypes appeared from CS14 up to CS19 (Fig. 5D), indicating delayed neurogenesis of the PNS system in comparison with the CNS. This suggests that the diversification and specialisation of neurons in the PNS occurs at later developmental stages than in the CNS.

### Dynamics of neurogenesis in the human spinal cord

**Fig. 6.**
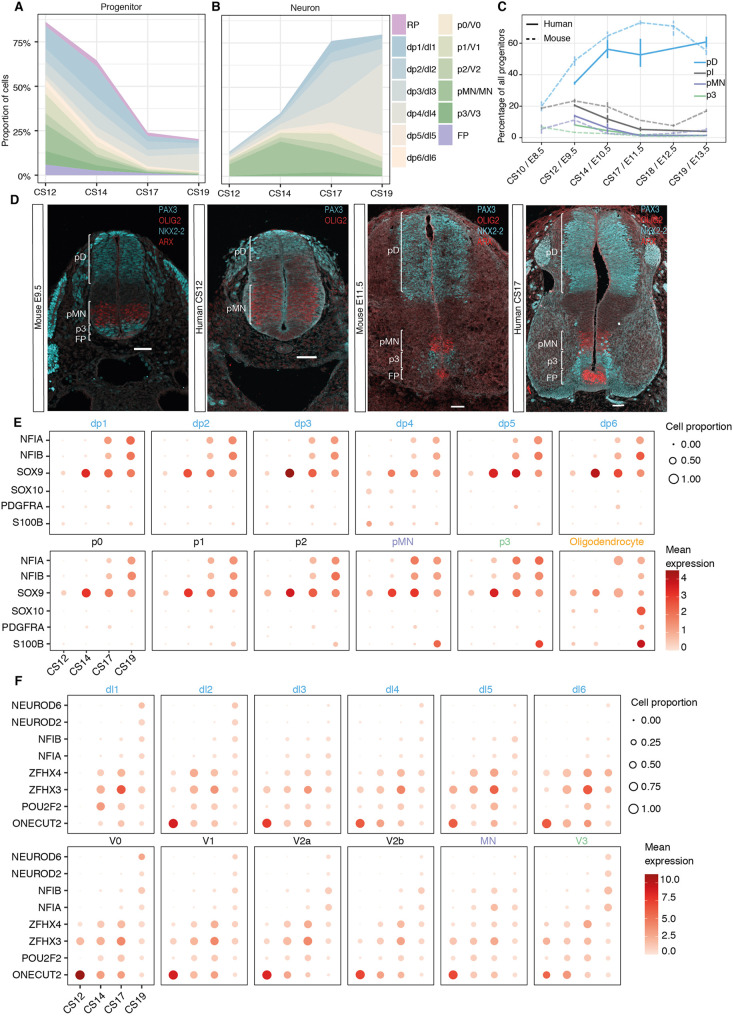
**Dynamics of progenitor and neuron proportions in the developing spinal cord.** (A,B) Proportions of progenitors and neurons in the spinal cord during gestational weeks 4 to 7. The data are proportional to the total neurons and progenitors at each time point. (C) Comparison of the ratio of progenitors between mouse and human grouped in broad territories: pD (dp1-dp6); pI (p0-p2). Vertical bars indicate the range around the mean of proportions per sample. (D) Immunofluorescence of PAX3 (cyan), OLIG2 (red), NKX2-2 (cyan) and ARX (red) in transverse sections of mouse and human cervical neural tube at E9.5 and E11.5 in mouse embryos and CS12 and CS17 in human embryos. Scale bars: 50 µm. (E) Expression of gliogenic markers in progenitors and oligodendrocytes. (F) Expression of the temporal transcription factor code in neurons. In E and F, the size of the circles indicates the proportion of cells that express the gene per stage and domain, and the colour indicates the mean expression levels.

Because the single-cell transcriptomic atlas of the mouse neural tube captured temporal changes in progenitor and neuronal populations (Delile et al., 2019), we performed a similar analysis in the human spinal cord. Despite the reduced temporal resolution of the dataset, with four different stages from a period of approximately 2 weeks, changes in the proportions of progenitors and neurons were evident (Figs 1B and 6A,B). The change in the relative proportions of pMN and p3 progenitors were consistent with the measured changes of progenitor domain sizes in the spinal cord over time in human embryos (Fig. 6A,B) (Rayon et al., 2020). Extending the analysis to all progenitors and neuronal subtypes revealed that at CS12 ventral progenitors and V1, V2, MN and V3 neurons were more abundant than dorsal subtypes. The relative abundance of neural progenitors decreased over time as neurons increased (Fig. 6A,B). Between CS14 and CS19, the generation of neurons in ventral domains appeared to slow (Fig. S6A). By contrast, the rate of neurogenesis in dorsal domains increased, with proportionally more dorsal than ventral neurons by CS19 (Fig. 6A,B). This is consistent with the dynamics of neuronal subtype differentiation observed in other vertebrates (Kicheva et al., 2014).

We next compared mouse and human spinal cord development by aligning the datasets using major developmental events observed in the two species (Rayon et al., 2020). We grouped the 11 neural progenitor domains into dorsal interneuron progenitors (pD; dp1-dp6), intermediate interneuron progenitors (pI; p0-p2), pMNs, and ventral interneuron progenitors p3. A broadly similar pattern of changes in the proportions of cell types were evident in mouse and human, although the tempo of human development was considerably slower than that of mouse. More detailed inspection indicated that in the CS12 embryo, the proportion of p3 and pMN ventral progenitors compared with pI and pD was higher in human than mouse (pD and pI human 55% cells, mouse 72% cells; pMN and p3 human 22% cells, mouse 16% cells; Table S5), which was also evident by immunostaining (Fig. 6D). The increased proportion of ventral progenitors persisted in the CS14 embryo (pMN and p3 human 11% cells, mouse 5% cells), but in the CS17 embryo the size of the ventral domains was comparable between mouse and human (pMN and p3 in human and mouse 3%) (Fig. 6C,D, Fig. S6B).

Next, we compared the ratio of neurons over progenitors across domains per time point as a proxy of the rate of neurogenesis. Whereas there was little neurogenesis between CS12 and CS14, the neuronal output increased in all domains from CS17 onwards (Fig. S6A). As expected, there was an earlier onset of neurogenesis in the mouse and human pMN than in the rest of the domains (Fig. S6) (Kicheva et al., 2014; Rayon et al., 2020), but the rate of MN differentiation in human persisted at a higher rate from CS17 onwards compared with mouse. In contrast, the rate of neurogenesis in the pD and pI domains was comparable between mouse and human across time points (Fig. S6). Neurogenesis in the pD domain in the CS17 and CS19 embryos appeared to be constant, whereas there was a marked increase in pD progenitors from E11.5 to E13.5 in mouse (Fig. S6), consistent with a slower rate of dorsal neurogenesis in human.

Together, the analysis indicated that, although mouse and human display an overall similar pattern of neurogenesis, the human neural tube has a higher initial proportion of pMN and p3 progenitors and pMN cells undergo a higher relative rate of neurogenesis. By contrast, dorsal neurogenesis may persist for a longer period in human than mouse. Further validation including more embryos spanning later time points will be needed to determine whether human neurogenesis is delayed.

Finally, we analysed the sequential expression of gliogenic markers in progenitors. *SOX9* was detected at low levels across progenitor domains from CS12, followed by the detection of *NFIA/B* at CS17 at higher levels in ventral progenitors, its expression increasing in dorsal progenitors by CS19 (Fig. 6E). The expression of *SOX9* and *NFIA/B* in progenitors and neurons was consistent with immunohistochemical assays of the embryonic human spinal cord (Betters et al., 2010; Dady et al., 2021 preprint; Deneen et al., 2006; Rayon et al., 2020). The expression of NFIA correlates with the onset of gliogenesis and is observed at CS15 in ventral progenitors but delayed until ∼CS18 in dorsal regions (Betters et al., 2010; Dady et al., 2021 preprint; Deneen et al., 2006; Rayon et al., 2020). In addition, we identified a small number of cells (*n*=77), present from CS12 onwards, expressing *SOX10*, *SOX9*, *PDGFRA* and *S100B*, characteristic of oligodendrocytes (Fig. 6E).

### A conserved temporal code for the specification of neuronal identity

In parallel to the spatial patterning of neurons, a temporal transcription factor code has been identified in mouse and is responsible for the further diversification of neuronal identity throughout the CNS (Sagner et al., 2020 preprint). To investigate whether this temporal code is conserved in humans, we examined the expression of the transcription factors that correlate with the early- and late-born neuronal classes in all DV domains. Consistent with analysis of gene expression in the mouse neural tube, human neurons present at the earliest time point (CS12) express Onecut-family members but little if any *POU2F2*, *ZFHX2-4*, *NFIA/B/X* or *NEUROD2/6*. By CS14, many neurons expressed *POU2F2* and *ZFHX2-4*, characteristic of neurons born at intermediate times in mouse. By contrast, expression of *NEUROD6*, *NEUROD2*, *NFIA* and *NFIB*, which identify later-generated neurons in mouse, were only detected in neurons by CS19. An exception was MNs, where NFI factors were detected from CS14 (Fig. 6F), similar to the earlier expression of these TFs in mouse MNs. The relatively sparse expression of late-born neuronal markers in dI3-dI6 in the CS19 sample suggests that neurogenesis in the human developing spinal cord continues at later time points, consistent with an extended neurogenic period in comparison with mouse. Collectively, these results indicate a conserved temporal code for the diversification of neurons that is temporally protracted in the dorsal portion of the spinal cord in human compared with mouse.

### Transcriptional dynamics during neurogenesis in mouse and human

Next, to reconstruct and compare gene expression dynamics during the differentiation of specific neuronal subtypes we used RNA velocity (La Manno et al., 2018) to analyse the temporal dynamics of gene expression. We inferred RNA velocity using scVelo (Bergen et al., 2020) and modelled neurogenic lineages with CellRank (Lange et al., 2020), which infers developmental trajectories from splicing information and transcriptional similarity. For the analysis, we included mouse samples from E9.5 to E13.5 and the CS12, CS14 and CS19 human datasets. Because these methods are very sensitive to the average counts per gene in each dataset, we excluded the CS17 sample, which has lower average gene counts than the other human samples (Fig. S7A).

**Fig. 7.**
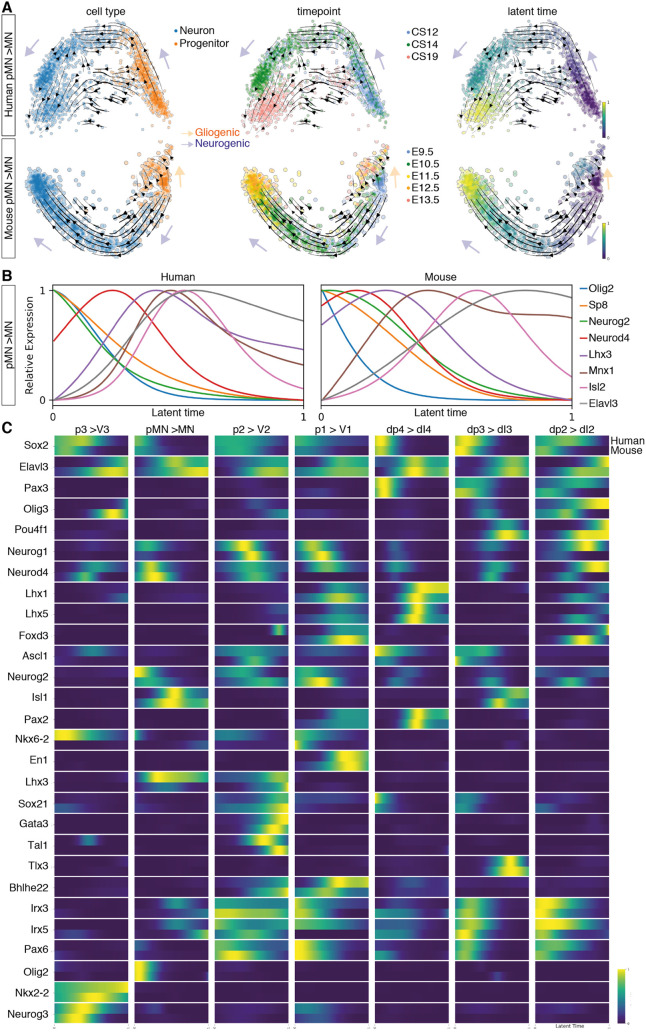
**Neurogenic trajectories in the developing spinal cord.** (A) PCA of the pMN-to-MN trajectory in human and mouse indicating the RNA-velocity trajectories depicted by black arrows in the PCA. PCAs are annotated by cell type (left), time point (middle) or latent time (right). The earliest latent time points are indicated in dark blue and latest in yellow on the latent time colour scale. Neurogenic trajectories are indicated with blue arrows, and the gliogenic trajectory in the mouse pMN is depicted by the orange arrow pointing away from the neurogenic trajectory. (B) Smoothed expression profile of the reconstructed motor neuron differentiation trajectory using the calculated latent times in human and mouse for selected genes. (C) Heatmap of the normalised gene expression (blue, low; yellow, high) of genes involved in neurogenesis for each neuronal class as a function of latent time in human (top bar) and mouse (bottom bar).

The inference of neurogenic trajectories from the mouse dataset was overall better resolved than in human, likely owing to the higher temporal resolution of the mouse datasets compared with the human. Nevertheless, reconstructing the differentiation of neurons of several DV domains in mouse and human (p3, pMN, p2, p1, dI4, dI3, dI2) showed single convergent neurogenesis paths from progenitors to neurons in each case (Fig. 7A, Fig. S7B). Moreover, the analysis highlighted a temporal trajectory within some progenitors, including p3, pMN and p2 in mouse (Fig. 7A, Fig. S7B), which is consistent with the temporal programme of gene expression in progenitors and the gliogenic switch in the spinal cord, where neural progenitors from E11.5 give rise to neurons and glia (Kang et al., 2012; Sagner et al., 2020 preprint).

Latent time estimations of the probability of a cell to reach the differentiated neuronal state allowed directional ordering along the trajectories and the inference of smoothed gene expression dynamics in latent time. In both mouse and human, the neurogenic trajectory from pMN to MN confirmed the expected sequential progression of gene expression during differentiation: progenitor genes, including Olig2, were expressed earlier in latent time, followed by neurogenic genes, such as Neurod4, and, finally, the markers of post-mitotic neurons Elavl3 and Mnx1 were detected at the latest latent time points (Fig. 7B). We then examined the gene expression dynamics for the differentiation of V3, V2, V1, dI4, dI3 and dI2 neurons in mouse and human (Fig. 7C, Fig. S7C). Heatmaps of the dynamics of gene expression in latent time were generally similar in the two species, and resembled the known dynamics of the genes as well as the pseudotemporal ordering observed in the mouse scRNA-seq (Fig. 7C) (Delile et al., 2019). Overall, these analyses suggested that the genetic programmes for the differentiation of neurons in the developing spinal cord are conserved in mammals.

### Co-expression of Olig2 and Nkx2-2 in the developing spinal cord

**Fig. 8.**
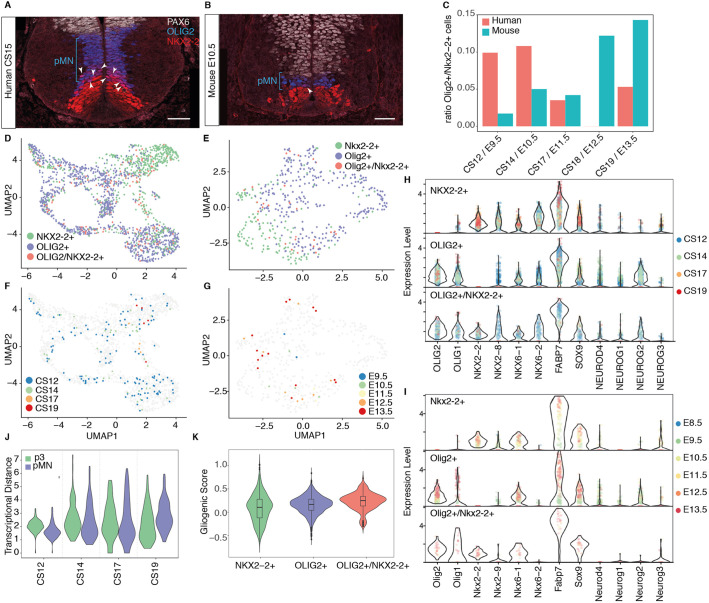
**Overlapping expression of OLIG2 and NKX2-2**. (A,B) Expression of the ventral progenitor markers PAX6 (white), OLIG2 (blue) and NKX2-2 (red) in transverse sections of human (A) and mouse (B) cervical neural tube at CS15 and E10.5, respectively. Scale bars: 50 µm. (C) Ratio of Olig2^+^/Nkx2-2^+^ double-positive cells within all cells expressing Nkx2-2 or Olig2. (D-G) Human UMAP of all cells expressing Nkx2-2 or Olig2 in human (D,F) and mouse (E,G). In F,G the double-positive progenitors are colour coded by time point. (H,I) Violin plots of selected genes involved in pMN and p3 neurogenesis and progenitor maturation in human (H) and mouse (I). Cells are labelled by time point. (J) Transcriptional distance of double-positive progenitors to pMN and p3 cells. Values closer to 1 indicate greater similarity to the population. (K) Gliogenic score of cells expressing *OLIG2*, *NKX2-2* or both genes in human.

At early stages of neural development in mouse and chick, pMN cells express OLIG2 but not NKX2-2 and give rise to MNs. This distinguishes pMN progenitors from NKX2-2-expressing p3 cells (Briscoe et al., 1999; Novitch et al., 2001). At later developmental stages, once progenitors finish generating MNs, oligodendrocyte precursor cells (OLPs) in the region of the mouse neural tube previously occupied by pMN cells co-express OLIG2 and NKX2-2 and produce oligodendrocytes (Richardson et al., 2000; Zhou et al., 2001). By contrast, in human embryos a substantial number of OLIG2 and NKX2-2 co-expressing cells are observed in the pMN domain at early developmental stages, when MNs are still being generated ((Marklund et al., 2014); Fig. 8A,B, Fig. S8A,B). These are thought to represent a pMN subdomain in human (Marklund et al., 2014). To define the molecular identity of OLIG2 and NKX2-2 co-expressing cells, we analysed Sox2^+^ progenitors that expressed either Nkx2-2 or Olig2 in mouse and human. In mouse, the number of *Olig2^+^/Nkx2-2*^+^ co-expressing cells comprised <5% of the total number of Nkx2-2- or Olig2-expressing cells until E11.5. By contrast, the proportion of *OLIG2*/*NKX2*-2 double-positive cells was ∼10% in equivalent staged human embryos (Fig. 8C, Fig. S8C,D). By E12.5 in mouse, at the onset of gliogenesis, the number of the *Olig2*^+^/*Nkx2-2^+^* co-expressing progenitors had increased to ∼15%, correlating with the appearance of OLPs in the developing spinal cord. This increase was not detected in human, where the ratio of *OLIG2*^+^/*NKX2-2^+^* co-expressing progenitors in human decreased at CS17 and CS19 (Fig. 6C).

To investigate whether Olig2^+^/Nkx2-2^+^ co-expressing cells had a distinct transcriptional signature, we compared Olig2^+^/Nkx2-2*^+^* cells with Olig2^+^/Nkx2-2^−^ pMN and Olig2^−^/Nkx2-2^+^ p3 progenitors. UMAPs of mouse or human cells showed that Olig2^+^/Nkx2-2*^+^* cells projected to regions of the embedding that contained either pMN or p3 cells (Fig. 8D,E). Olig2^+^/Nkx2-2^+^ cells failed to cluster according to developmental stage (Fig. 8F,G). Moreover, analysis of pMN and p3 markers in human and mouse Olig2^+/^Nkx2-2^+^ cells showed a similar transcriptional signature, which closely resembled a combination of the pMN and p3 signatures (Fig. 8H,I). To determine the similarity of Olig2^+^/Nkx2-2^+^ cells to either pMN cells or p3 cells, we averaged the expression profiles for each cell type per time point and calculated the distance between double-positive cells and pMN or p3 cells in human. This suggested that double-positive cells were somewhat closer in gene expression space to pMN cells at CS12 and CS14 than to p3 cells (Fig. 8J), but the degree of similarity with p3 cells increased at later time points (Fig. 8J). Together, this analysis suggests that human *OLIG2^+^/NKX2-2^+^* co-expressing progenitors resemble an amalgamation of pMN and p3 identities.

The co-expression of OLIG2 and NKX2-2 in mouse and chick is indicative of OLPs. For this reason, the *OLIG2^+^/NKX2-2^+^* cells in human have been proposed to be OLPs (Marklund et al., 2014). Consistent with this, human *OLIG2^+^/NKX2-2^+^* cells expressed *FABP7*, *OLIG1* and *OLIG2*, characteristic of gliogenic progenitors, like mouse *Olig2^+^/Nkx2-2^+^* cells from older embryos (Fig. 8H,I). Whereas the expression of gliogenic markers in *Olig2^+^/Nkx2-2^+^* cells from mouse at early time points was low, the levels of *FABP7*, *OLIG1* and *OLIG2* in human double-positive progenitors were consistently high across time points (Fig. 8H). Likewise, we observed an increased gliogenic score in the double-positive progenitors compared with pMN and p3 progenitor populations (Fig. 8K). Taken together, these results are consistent with Olig2^+^/Nkx2-2^+^ cells representing OLPs. This would indicate that OLPs are more abundant at earlier developmental stages in the human neural tube than at the equivalent mouse stages, representing another example of heterochronic differences between rodents and primates. In chick and mouse, OLPs appear to arise mainly after pMN progenitors have ended MN generation, but the lineage relationship and the neurogenic or gliogenic potential of individual cells needs clarification (Leber and Sanes, 1995; Leber et al., 1990; Park et al., 2002; Richardson et al., 2000; Wu et al., 2006). In zebrafish, MNs and oligodendrocytes have been proposed to arise from distinct cell lineages that initiate *Olig2* expression at different times (Ravanelli and Appel, 2015). It will be important to establish the lineage relationships between pMNs, MNs and OLPs in human, and establish the neurogenic or gliogenic potential of double-positive cells.

### Online access to mouse and human neural tube transcriptome data

The availability of mouse and human transcriptome data from the developing neural tube and dorsal root ganglia provides a resource for developmental biologists studying the embryonic nervous system and for stem cell biologists aiming to refine differentiation protocols for the generation of specific neuronal subtypes. The *in vivo* characterisation of cell diversity in mouse and human will help identify the neuronal subtypes involved in locomotor circuits and provides a foundation to investigate subtype-specific and species-specific features that control connectivity and function. To provide access to both the human and mouse transcriptome data, we have established an interactive web application (https://shiny.crick.ac.uk/scviewer/neuraltube/). This will allow rapid and easy investigation of the expression of specific genes in all datasets described in this analysis (see Movie 1).

## MATERIALS AND METHODS

### Sample processing

Human embryonic material (gestational weeks 4-7) was obtained from the MRC/Wellcome Trust-funded (grant #006237/1) Human Developmental Biology Resource (HDBR57; http://www.hdbr.org) with appropriate maternal written consent and approval from the London Fulham Research Ethics Committee (18/LO/0822) and the Newcastle and North Tyneside NHS Health Authority Joint Ethics Committee (08/H0906/21+5). HDBR is regulated by the UK Human Tissue Authority (HTA; www.hta.gov.uk) and operates in accordance with the relevant HTA Codes of Practice. Embryos were freshly collected on Hibernate-E medium (A12486, Gibco) supplemented with 2% B-27 (17504001 Thermo Fisher Scientific) and 2.5 ml/l Glutamax (3505006, Gibco) and kept on ice until further dissection.

Trunk samples of single embryos were dissected as described by Delile et al. (2019). Briefly, samples were placed in Hanks Balanced Solution without calcium and magnesium (HBSS, 14185045, Life Technologies) supplemented with 5% heat-inactivated foetal bovine serum (FBS). For neural tube and dorsal root ganglia dissection, the superficial layers of skin, limbs and remaining organs were removed, whereas the somites, cartilage primordium, dorsal root ganglia and neural tube were left intact. Dissected samples were snipped into smaller pieces and incubated on a FACSmax (Amsbio, T200100) cell dissociation solution containing 10× Papain (30 U/mg, Sigma-Aldrich, 10108014001) for 11 min at 37°C to dissociate the cells and transferred to a HBSS solution with 5% FBS, Rock inhibitor (10 μM, Stemcell Technologies, Y-27632) and 1× non-essential amino acids solution (Thermo Fisher Scientific, 11140035). Single cells were disaggregated by pipetting and filtration, and quality control was assayed by measuring live cells versus cell death, cell size and number of clumps on an EVE cell counter (NanoEntek). Each sample was prepared at a concentration of 600-1700 cells/µl and viability ranged between 70 and 96%. Samples with a viability above 70% were used for sequencing.

### Single-cell RNA sequencing

Single-cell suspensions were loaded for each sample into a separate channel of a Chromium Chip G for use in the 10x Chromium Controller (PN-1000120). The cells were partitioned into nanolitre scale gel beads in emulsions (GEMs) and lysed using the 10x Genomics Single Cell 3′ Chip V3.1 GEM, Library and Gel Bead Kit (PN-1000121). The v2 kit was used for the E8.5 mouse as described by Delile et al. (2019). cDNA synthesis and library construction were performed as per the manufacturer's instructions. Libraries were prepared from 10 µl of the cDNA and 12 cycles of amplification. Each library was prepared using Single Index Kit T Set A (PN-1000213) and sequenced on the HiSeq4000 system (Illumina) using the configuration 28-8-98 on a single-index-paired-end run. Libraries were sequenced on independent flow cells for each sample and split across multiple lanes.

### Quality control and filtering

Gene expression was quantified from FastQ files using Cell Ranger (3.1.0) and the GRCh38-3.0.0 and mm10-3.0.0 indexes for human and mouse, respectively. A counts matrix was generated for each library using Cell Ranger count, submitting all technical replicates of a library together.

Putative cells reported by Cell Ranger in the filtered output matrix were inspected manually to remove droplets that reported an extremely high or low number of genes or UMI or had a high proportion of mitochondrial UMI. The thresholds applied to each replicate can be found in Table S1.

### Cell cycle and gene module scoring

Each cell was assigned a score based on the expression of a pre-determined list of cell-cycle gene markers (cc.genes, supplied with Seurat) with the CellCycleScoring function implemented in Seurat (Stuart et al., 2019). Mouse gene names were converted from human using biomaRt Ensembl-release 93 gene annotations. Additionally, gene module scores were calculated using the AddModuleScore function in Seurat for each gene module described in Table S2. Gene module scores were used for visualisation purposes on reduced dimension plots and to identify clusters of cells that were likely non-neural cells, which were removed from further analysis. The gliogenic score of *OLIG2*^+^/*NKX2-2*^+^ double-positive cells in human was determined using Seurat's AddModuleScore function with a set of known gliogenic markers (*FABP7*, *SOX9*, *SOX10*, *PDGFRA*, *CSPG4*, *FGFR3*, *FGFBP3*, *DBI*, *SLC1A3*, *HOPX*, *ALDH1L1*).

### Normalisation, integration and dimension reduction

Replicate datasets were subsequently filtered to remove contaminant cells (such as mesoderm and blood). The resulting datasets were then normalised and integrated using the SCTransform/IntegrateData workflow implemented in Seurat 3.2.2 (Stuart et al., 2019). For SCTransform, 3000 variable features were selected and cell cycle effects regressed using the difference in cell cycle scores (S-G2M). Replicates were then integrated together to create the time point and species datasets.

Principal components of integrated datasets were calculated using RunPCA with 3000 variable features to provide the first 70 components. UMAP and tSNE reductions were calculated using the RunUMAP and RunTSNE using the first 40 principal components.

### Cell type classification

We classified cells using Antler (https://juliendelile.github.io/Antler/) as described by Delile et al. (2019). Briefly, cells were classified based on the expression of known marker genes in a two-step process that first identified broad cell types before classifying neurons and progenitors further into 12 and 13 DV subtypes, respectively. The knowledge matrices (Tables S2 and S3) were adapted from Delile et al. (2019), converting mouse to human gene identifiers and replacing *Tubb3* with *STMN2* and *MAP2*, as *TUBB3* levels were low in human. In the human, additional marker genes were added for the PNS classification and for the identification of oligodendrocytes (Table S2).

### Mouse and human correlation analysis

First, a set of orthologous genes with a 1:1 correspondence was sought between human and mouse using Ensembl biomaRt and release-93 annotations. Gene sets were then defined. The ‘transcription factor’ gene set was defined as the unique set of genes with which the GO term ‘GO:0003700’ was associated, using human biomaRt connection.

Expression matrices were subset by cell type and gene set. Human gene names were converted to mouse gene names and genes with no 1:1 homologue were discarded. Representative expression profiles per cell type were calculated using the mean gene expression. Each representative profile was correlated using the ‘cor.test’ function in R and the Pearson method.

### Comparison with bulk *in vitro* RNA-seq data

Data from ventral (p3, pMN, p2, p1, p0) domain progenitors and neurons were aggregated and averaged per time point. A subset of 1448 highly expressed genes with varying dynamics along the neurogenic differentiation trajectory was chosen to compare this pseudo-bulk data with bulk *in vitro* data published previously (Rayon et al., 2020). Data from each time point was normalised between 0 and 1 to improve comparability and Spearman's correlation coefficient across all average expressions was calculated between each *in vivo* and *in vitro* time point.

### Correlation analysis for *PAX7*

To investigate *PAX7* expression in FP cells, Spearman's correlation coefficient was used to select the 30 genes that had the strongest positive correlation with *PAX7* in cells annotated as FP. Mean expression levels of the genes in human FP cells, pD cells and mouse FP can be found in Table S4.

### RNA velocity

Spliced/unspliced count matrices were generated from aligned reads using the Velocyto (La Manno et al., 2018) command line interface tool with the run10x command and default arguments. For reference genomes and repeat masks, GRCh38-3.0.0 and mm10-3.0.0 were used for human and mouse, respectively.

Only cells with Antler classifications were used for downstream analysis without the SCTransform/IntegrateData workflow implemented in the Seurat 3.2.2 normalisation step (Stuart et al., 2019). It was noted that CS17 data had lower counts per gene than other time points and this produced artefacts in normalisation and scaling pre-processing steps for principal components analysis (PCA) (Fig. S6A). As a result, CS17 was not included in the trajectory analysis. Mouse E8.5 data were also excluded as there was no corresponding time point in human data. Initial inspection of p0, dl6, dl5 and dl1 domains revealed an insufficient number of cells to model the progenitor-to-neuron transition and so these domains were not included in further trajectory analysis.

For downstream analysis, Scanpy was used to normalise and log transform the data. To divide genes into subsets, 2000 highly variable genes were selected for each time point separately, and genes common to all time points were retained. This was done to reduce batch effects between time points. To these highly variable genes, a curated list of 177 genes known to be involved in neural tube development was added. Gene selection, PCA and velocity modelling were performed on each domain independently. scVelo (Bergen et al., 2020) was used to recover ‘latent time’ dynamics, infer velocities on ‘stochastic’ mode, and embed these velocities in PCA space.

### CellRank trajectory analysis

CellRank (Lange et al., 2020) was used to construct neurogenic gene trends. CellRank models cellular dynamics as a Markov chain whereby transition probabilities are calculated using both RNA velocity and transcriptomic similarity. First, each domain's terminal states were inferred with the number of terminal states fixed to either two or one depending on whether a bifurcation of progenitor fates was visible in the first two PCA components (for both mouse and human: two terminal states for p3, pMN, p2, p1 and dl2, one for dl4 and dl3). The weight_connectivities parameter, which determines the relative importance of velocity and transcriptomic similarity for terminal state calculation, was adjusted for each domain to achieve a realistic prediction (for p3, pMN, p2, p1, dl4, dl3, dl2: 0.2, 0.7, 0.7, 0.2, 0.7, 0.9, 0.5, respectively, in human and 0.2, 0.2, 0.3, 0.2, 0.7, 0.7, 0.5, respectively, in mouse). To produce gene trends, a generalised additive model (GAM) was fitted to each gene's expression pattern through latent time using CellRank's ul.models.GAM function. This was done with 5 knots and a spline order of 2. This function weights a cell's contribution to the model by its lineage probability, so progenitors committing to a gliogenic/late progenitor identity did not contribute to neurogenic gene trends. If this GAM failed to fit to a gene's expression profile, or the GAM's confidence interval achieved a range >10, the expression profile was fixed to zero.

### Immunostaining and microscopy

Immunohistochemistry on human and mouse spinal cord tissues and on mouse and human cells was performed as described previously (Rayon et al., 2020). Rabbit anti-PAX6 (Covance, PRB-278P-100, 1:500), goat-anti OLIG2 (R&D Systems, AF2418, 1:800), mouse anti-NKX2-2 (BD Pharmigen, 74.5A5,1:500), mouse anti-NKX6-1 [Developmental Studies Hybridoma Bank (DHSB), F55A10, 1:100], mouse anti-PAX3 (DSHB, clone C2, 1:100), rabbit anti-OLIG2 (Millipore, AB9610, 1:1000), rabbit anti-ARX (Poirier et al., 2004; 1:1000) and guinea pig anti-NKX6-2 (Vallstedt et al., 2001; 1:8000) were used. The anti-NKX6-2 antibody raised against the 11 amino acid N-terminal mouse epitope contained a mismatch [alanine (A)>threonine (T)] in the human sequence and did not detect NKX6-2 in human samples. Three embryos per stage were used for mouse. In human, three embryos were used for PAX6, OLIG2 and NKX2-2, and three sections of a single human embryo per stage were used for PAX3, OLIG2, NKX2-2 and ARX immunostaining.

Cryosections were imaged using a Leica SP8 confocal microscope equipped with a 20× NA 0.75 dry objective, or a Leica SP5 confocal microscope. *Z*-stacks were acquired and represented as maximum intensity projections using ImageJ software. Pixel intensities were adjusted across the entire image in Fiji. The same settings were applied to all images.

### RNAscope FISH with immunohistochemistry

Transverse cryosections (14 µm thickness) were cut onto Superfrost Plus slides (Thermo Scientific, 10149870) as described by Rayon et al. (2020). Slides were stored at −80°C until ready to be processed. The RNAscope multiplex fluorescent v2 kit was used as per the manufacturer's instructions for fixed frozen samples (ACD Bio), with the protease treatment step shortened to 15 min. The RNAscope probe Hs-NKX6-2 (48674) was used. At the end of the RNAscope protocol, sections were fixed in 4% paraformaldehyde for 15 min at room temperature and then washed twice in 1× PBS for 5 min. Sections were incubated in blocking solution (1% bovine serum albumin, 0.1% Triton X-100 in 1× PBS) for 30 min at room temperature and then incubated in primary antibody (DHSB, F55A10, 1:100) in blocking solution 1 h at 4°C. Sections were then washed three times for 5 min each in 1× PBS, incubated with secondary antibody (donkey anti-mouse Alexa Fluor 488 conjugate; Invitrogen, A32766, 1:1000) for 30 min at room temperature, rinsed in 1× PBS three times for 5 min each, and mounted with ProLong Gold with DAPI mounting medium (Thermo Fisher Scientific). Sections were imaged using a 40× oil immersion lens on a Leica SP8 confocal microscope. Images were taken with the pinhole set to 2 AU. Three sections of a single human embryo were recorded per stage.

### Image analyses of mouse and human spinal cord sections

The mean fluorescence intensity in immunostained sections was quantified across a line adjacent to the apical lumen employing a Fiji macro described by Zagorski and Kicheva (2018). For the analysis, three mouse embryos per stage and three sections from single human embryos were used. Data were plotted in R.

## Supplementary Material

Supplementary information

Reviewer comments
