# Supplementary material for: Single-cell transcriptome profiling of the human developing spinal cord reveals a conserved genetic programme with human-specific features
Source: Development. 2021 Aug 5;148(15):dev199711. doi: 10.1242/dev.199711 (PMC8353162; doi:10.1242/dev.199711)
Supplement: Supplementary information [file develop-148-199711-s1.pdf]

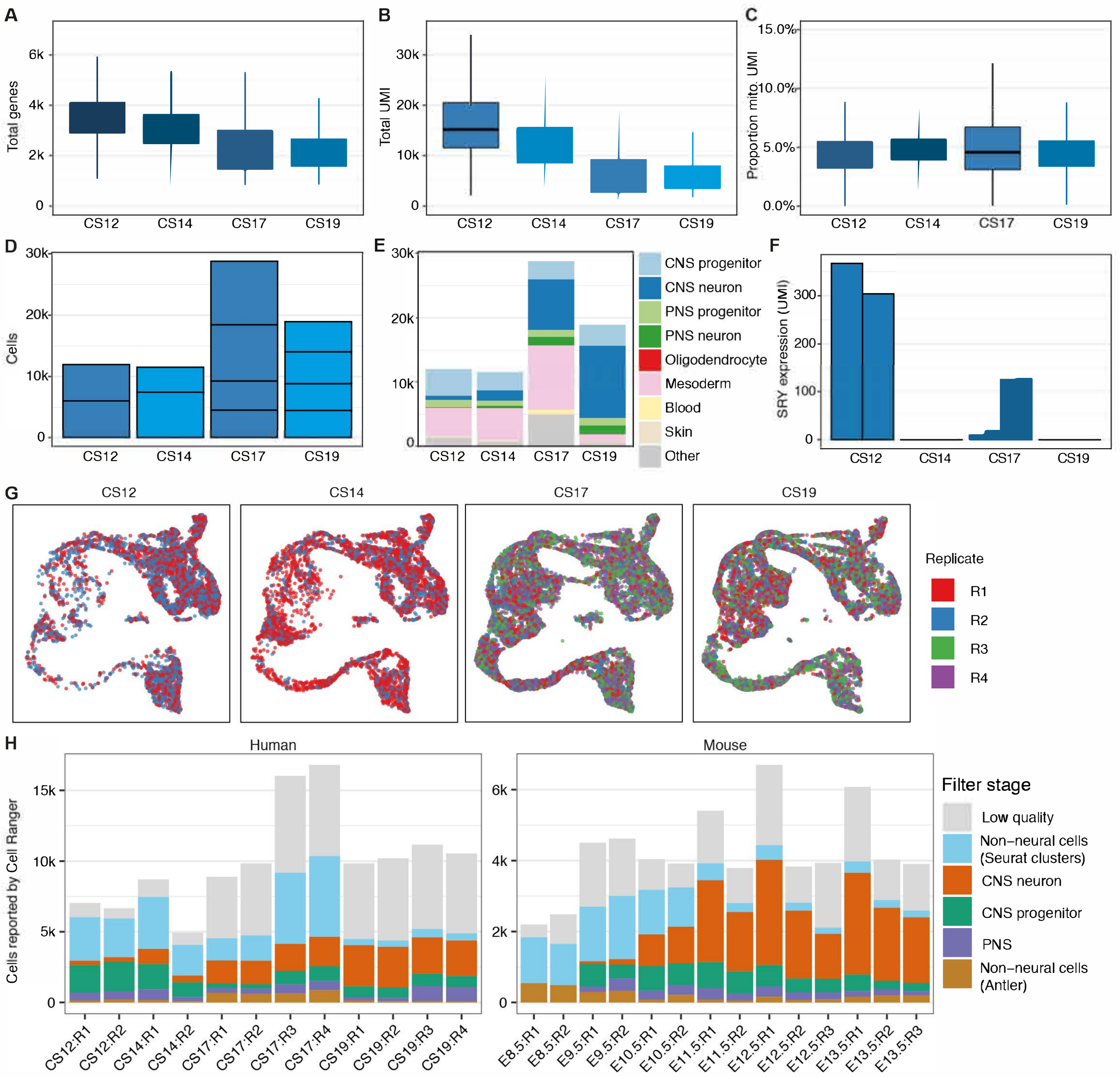

Fig. S1. Characterization of the single cell transcriptome dataset.
(A) Number of genes per cell for each stage. **(B)** Number of unique molecular identifiers (UMI) per cell for each stage. **(C)** Proportion of mitochondria UMI per cell for each stage. **(D)** Number of cells per replicate in each timepoint. **(E)** Number of cells classified per cell type in each stage. **(F)** Expression of the male-specific gene SRY per embryo. **(G)** UMAP of neural cells by timepoint and replicate. **(H)** Number of cells identified by Cell Ranger for each embryo. Low quality cells were removed using (A-C). Curated cells were classified with Seurat and Antler.

Development • Supplementary information

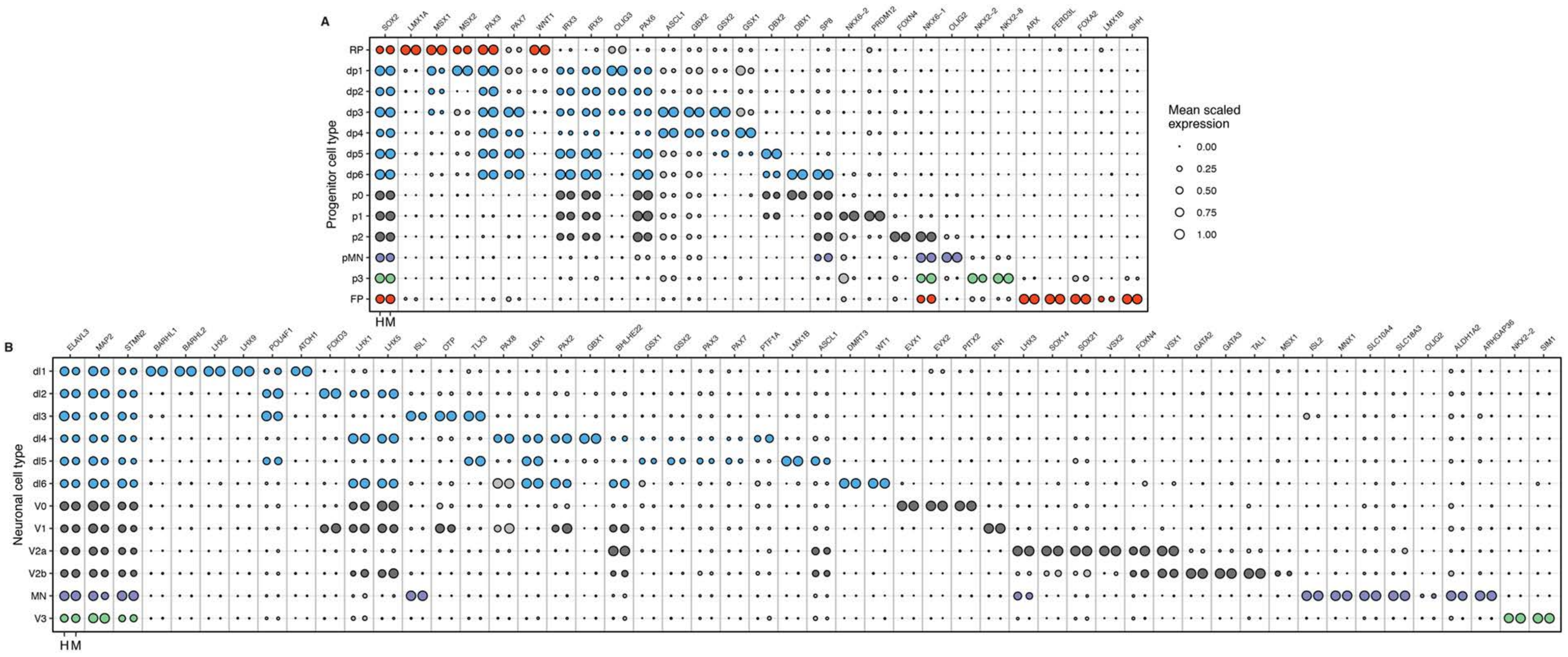

Fig. S2. Classification of dorsoventral progenitors and neuronal classes in human and mouse.
(A, B) Combined bubble plot that depicts the expression of markers used to identify DV domains in human and mouse **(A)** progenitors and **(B)** neurons. Genes chosen for cell assignment are coloured; grey circles correspond to markers not used for the selection of a specific population. Circle size indicates mean scaled gene expression levels.

Development • Supplementary information

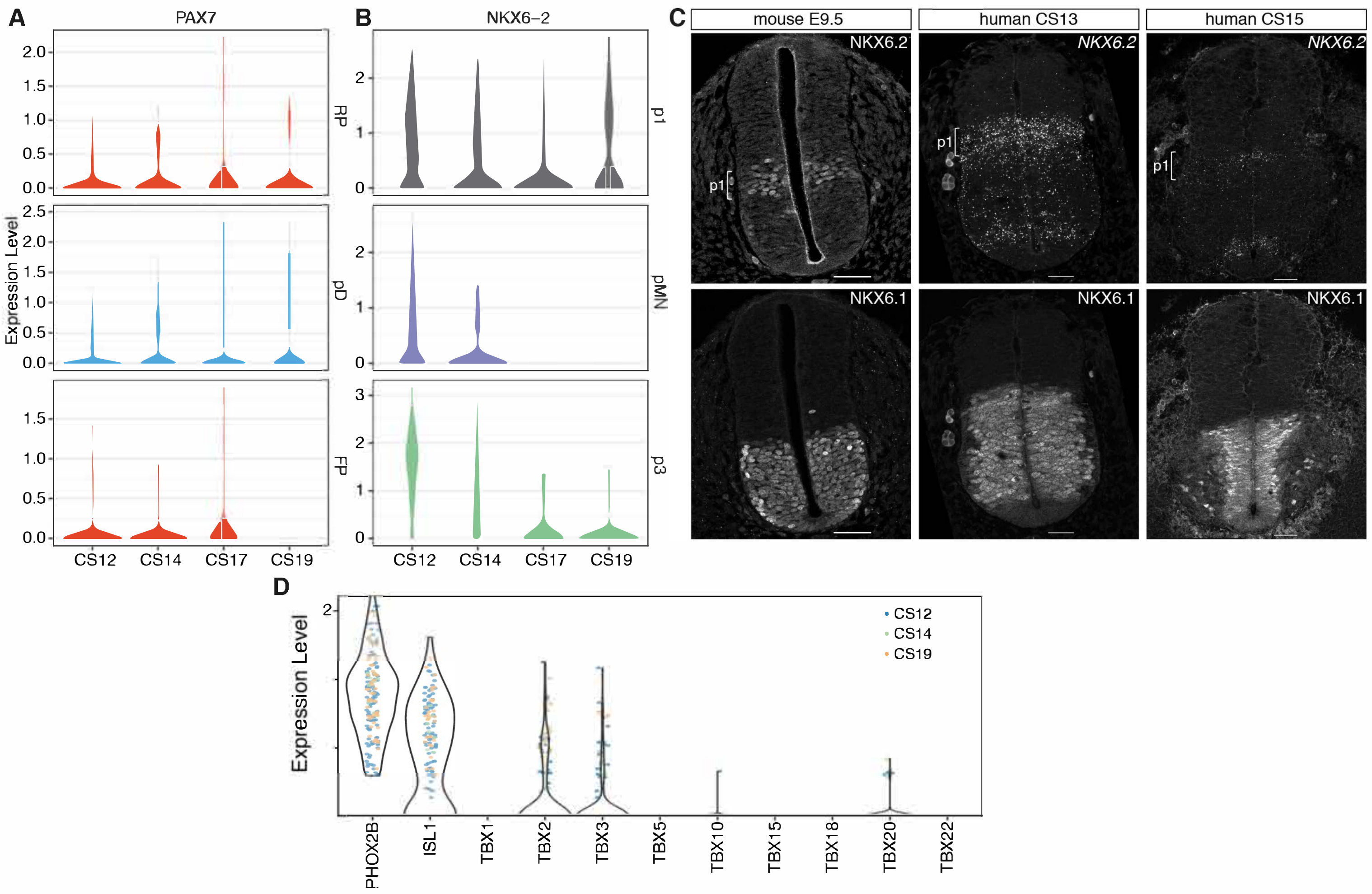

Fig. S3. Further characterization of human-specific features of neural progenitors and visceral motor neurons.
(A) Violin plots of PAX? expression in the floor plate (FP), pD (dp1-dp6) and roof plate (RP) at the indicated timepoints. **(B)** Violin plots of NKX6.2 expression in p1, pMN and p3 progenitors. **(C)** NKX6.2 and NKX6.1 expression in transverse sections of the mouse and human neural tube at shoulder levels in mouse E9.5, and human CS13 and CS15 embryos. Scale bars, 50µm. **(D)** Violin plots for the expression of the indicated TBX genes in visceral motor neurons classified according to the expression of PHOX2B in human color coded by timepoint.

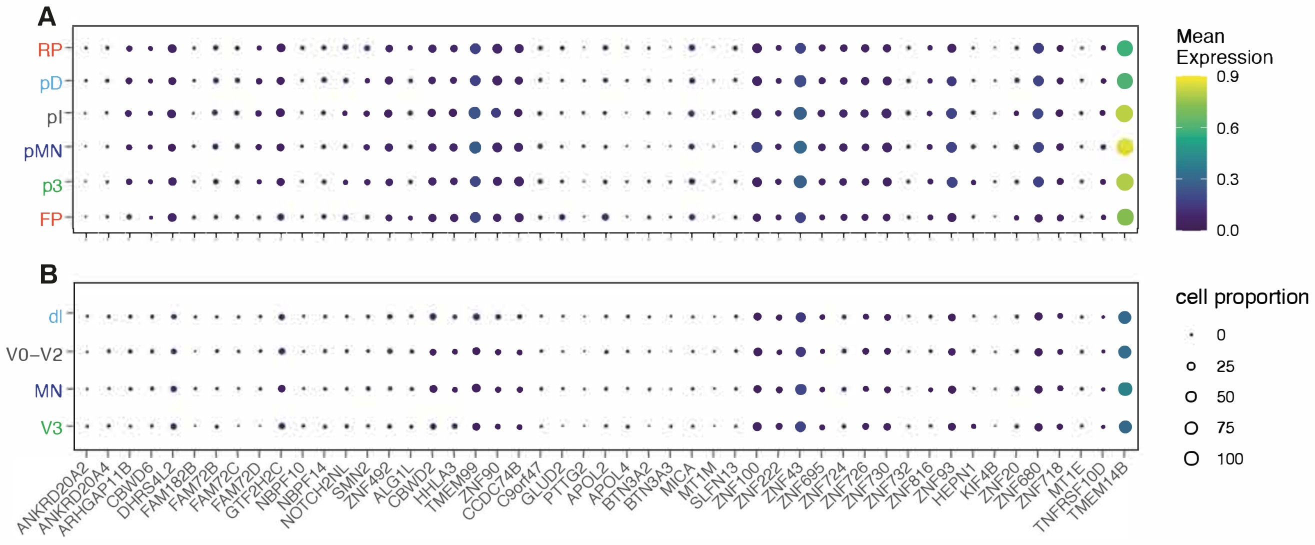

Fig. S4. Expression of primate-specific genes in progenitors and neurons of the human developing spinal cord.

(A, B) Bubble plots that depict the expression of primate specific genes in **(A)** DV progenitors or **(B)** neurons. The size of the circles indicates the proportion of cells that express the gene per stage and domain, and the colour indicates the mean expression levels. pD (dp1-dp6), pI (p0-p2), dI (dI1-dI6).

Development • Supplementary information

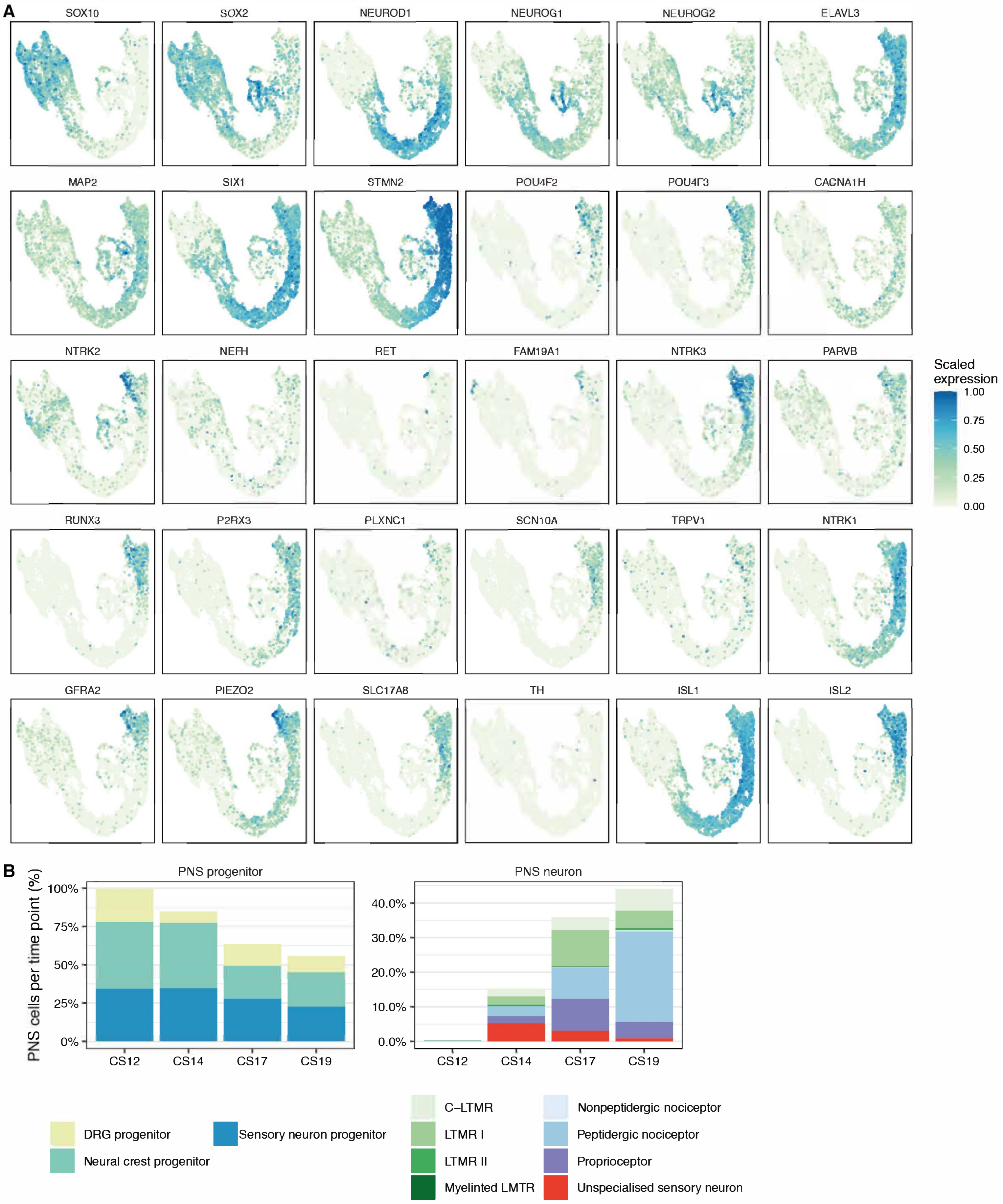

Fig. S5. Further classification of peripheral nervous system cells in the developing human embryo.
(A) Feature plots of genes selected for the classification. (B) Proportion of peripheral nervous system (PNS) progenitors and neurons per time point.

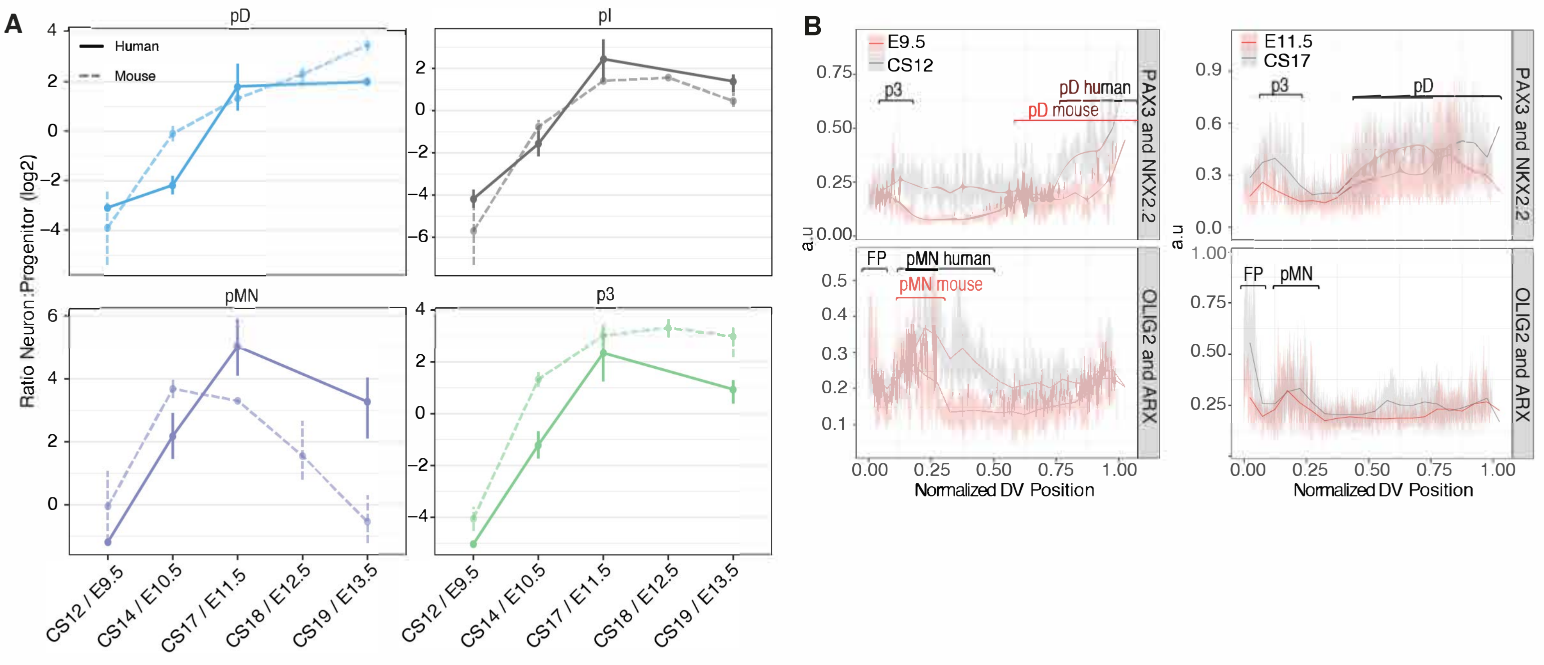

Fig. S6. Rate of neurogenesis and domain sizes in the developing spinal cord.
(A) Ratio of neurons to progenitors per domain across timepoints in mouse and human grouped in broad territories: pD (dp1-dp6); pI (p0-p2). Vertical bars indicate the range around the mean of proportions per sample. **(B)** Quantification of the mean fluorescence intensity in immunostained sections to determine domain sizes in mouse and human (mouse n= 3 embryos per stage, human n=1 embryo per stage using 3 sections).

Development • Supplementary information

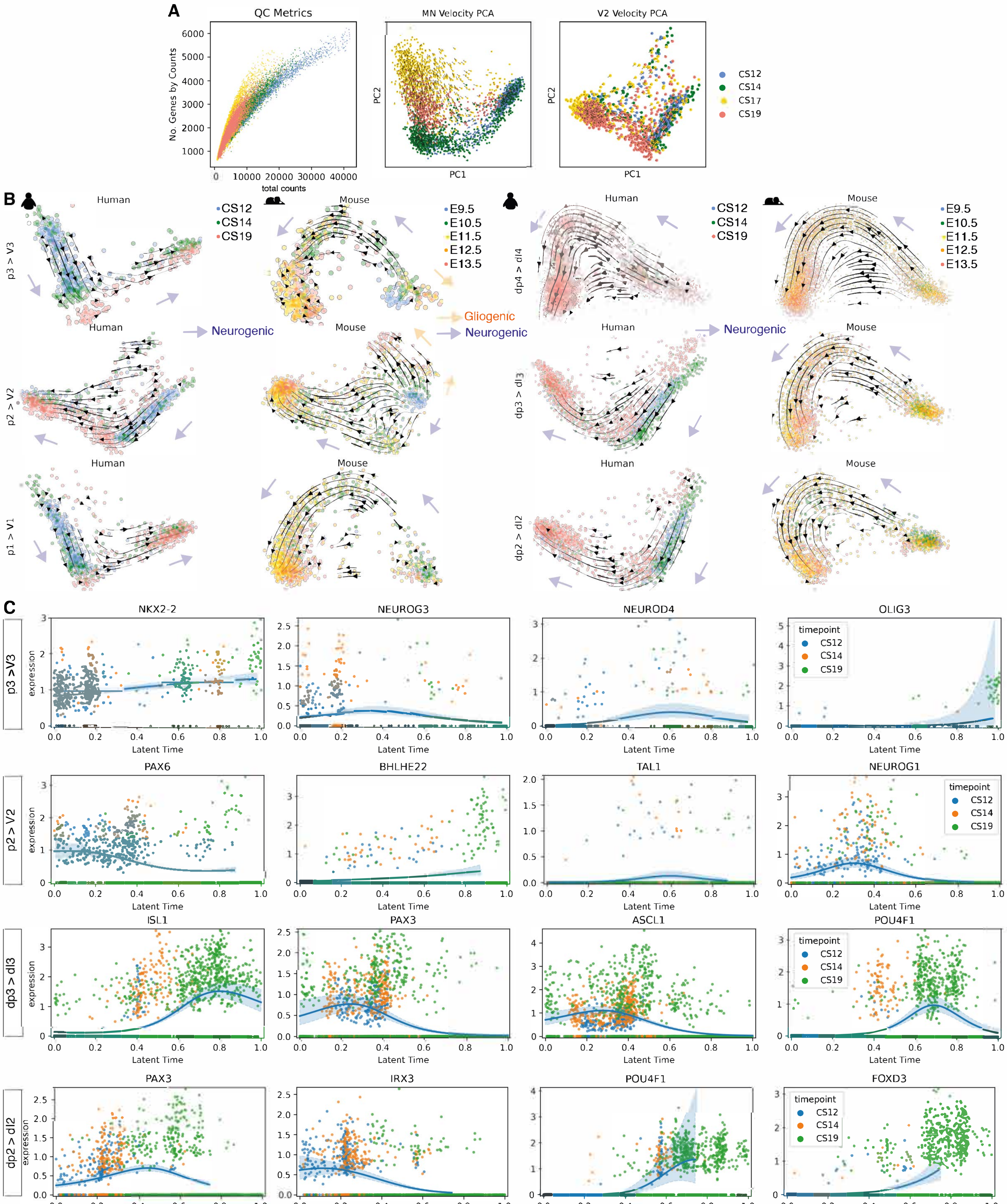

Fig. S7. Neurogenic trajectory interference across dorsoventral domains of the developing spinal cord.
(A) Total counts per number of genes in each timepoint for velocity analysis and PCA of the pMN to MN trajectory in human (middle) and the PCA of the V2 trajectory including the CS17 sample. **(B)** PCA of neurogenic trajectories in human and mouse indicating the RNA-velocity trajectories depicted by black arrows in the PCA for dI2, dI3, dI4, V1, V2 and V3 neurons. PCAs colored by timepoint. Light arrows indicate the inferred trajectory paths. Neurogenic trajectories are indicated with blue arrows, and the gliogenic trajectories in the mouse p3 and p2 are labeled with orange arrows. **(C)** Gene expression levels of selected genes per cell along the estimated latent time with the interpolated smoothed expression in human. Cells colored by timepoint.

Development • Supplementary information

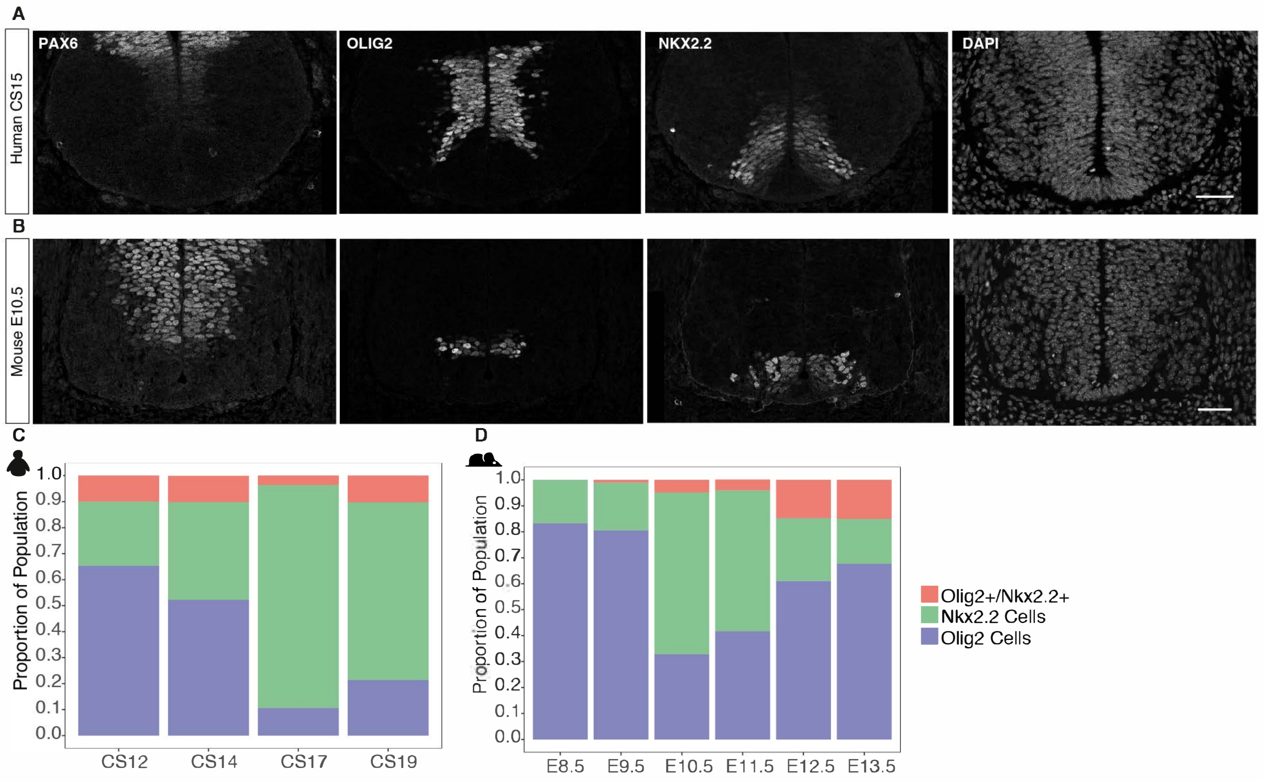

Fig. S8. Expression of Olig2 and Nkx2.2 in mouse and human.
(A, B) Expression of ventral progenitor markers PAX6, OLIG2, and NKX2.2 and DAPI in transverse sections of **(A)** human and **(B)** mouse cervical neural tube at CS15 and E10.5 respectively. Scale bars, SOμm. **(C, D)** Proportion of Olig2, Nkx2.2 and co-expressing cells across timepoints in **(C)** human and **(D)** mouse.

Table S1. Cell numbers and thresholds applied for each human sample.

Click here to download Table S1

Table S2. Knowledge matrix used to identify cell types in human.

Click here to download Table S2

Table S3. Knowledge matrix used to identify cell types in mouse (Delile et al., 2019).

Click here to download Table S3

Table S4. Mean expression levels of genes correlating with PAX7 expression in human FP, pD and mouse FP.

Click here to download Table S4

Table S5. Proportions of pD, pI, pMN and p3 progenitors across mouse and human timepoints.

Click here to download Table S5

Development • Supplementary information

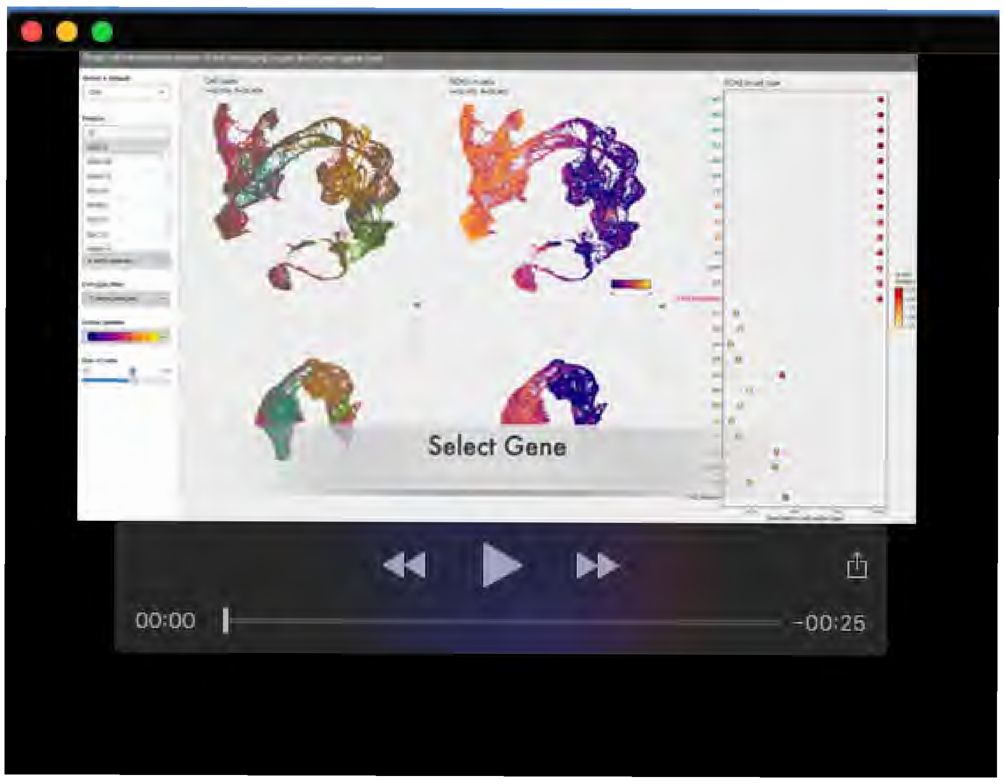

Movie 1. An interactive web application of the human and mouse transcriptome data.

Development • Supplementary information
