# Supplementary material for: Single-cell transcriptome profiling of the human developing spinal cord reveals a conserved genetic programme with human-specific features
Source: Development. 2021 Aug 5;148(15):dev199711. doi: 10.1242/dev.199711 (PMC8353162; doi:10.1242/dev.199711)
Supplement: Reviewer comments [file develop-148-199711_review_history.pdf]

Single cell transcriptome profiling of the human developing spinal cord reveals a conserved genetic programme with human-specific features

Teresa Rayon, Rory J Maizels, Christopher Barrington and James Briscoe
DOI: 10.1242/dev.199711

Editor: Samantha Morris

Review timeline

Original submission:	12 April 2021
Editorial decision:	19 May 2021
First revision received:	25 June 2021
Accepted:	5 July 2021

Original submission

First decision letter

MS ID#: DEVELOP/2021/199711

MS TITLE: Single cell transcriptome profiling of the human developing spinal cord reveals a conserved genetic programme with human specific features

AUTHORS: Teresa Rayon, Rory J Maizels, Christopher Barrington, and James Briscoe

I have now received all the referees reports on the above manuscript, and have reached a decision. The referees' comments are appended below, or you can access them online: please go to BenchPress and click on the 'Manuscripts with Decisions' queue in the Author Area.

The overall evaluation is positive and we would like to publish a revised manuscript in Development, provided that the referees' comments can be satisfactorily addressed. Please attend to all of the reviewers' comments in your revised manuscript and detail them in your point-by-point response. If you do not agree with any of their criticisms or suggestions explain clearly why this is so.

We are aware that you may currently be unable to access the lab to undertake experimental revisions. If it would be helpful, we encourage you to contact us to discuss your revision in greater detail. Please send us a point-by-point response indicating where you are able to address concerns raised (either experimentally or by changes to the text) and where you will not be able to do so within the normal timeframe of a revision. We will then provide further guidance. Please also note that we are happy to extend revision timeframes as necessary.

Reviewer 1

Advance summary and potential significance to field

Rayon and colleagues report the transcriptomic profiling of the cells that make up the developing human neural tube from gestational week 4 to 7 (CS 12, CS14, CS17 and CS19). A lot is known about spinal cord development in other vertebrates, but we know surprisingly little about human spinal cord development, mostly due to the difficulty to access tissue from human embryos. This is the

© 2021. Published by The Company of Biologists under the terms of the Creative Commons Attribution License (https://creativecommons.org/licenses/by/4.0/).

first single-cell atlas of the developing human neural tube to date and will be a tremendous resource for the community.

Rayon and colleagues use single-cell RNA-sequencing to identify progenitor and neuronal cell types of the central and peripheral nervous system of the human neural tube. They go on to compare these cell types with those previously identified in the mouse neural tube at equivalent developmental stages (Delile 2019 Development). Human and mouse cell types seem to share most of their transcriptional signature, however, Rayon and colleagues found intriguing differences too. For example, having transcriptomic insight into OLIG2+/NKX2.2+ cells in the human neural tube allowed the authors to propose that these cells are likely to be oligodendrocyte precursor cells and that oligodendrogenesis may start earlier in humans than in mice. The authors also make use of RNA velocity to infer the developmental trajectories of different neural progenitors as they give rise to neurons, and associated changes in gene expression. This type of analysis can inform strategies for in vitro generation of specific neuronal subtypes and/or help fine-tune existing protocols.

Besides providing the most detailed insight into human spinal cord cells, Rayon and colleagues have done a great job at making the data and the code accessible to everybody. Their single cell portal (https://shiny.crick.ac.uk/scviewer/neuraltube/) works beautifully and will become a reference for the community.

The only limitation of the paper is only having cells from one embryo for each stage. Most of my comments below relate to this, as I think that the authors should keep this in mind when making some conclusions. The small sample size is more than understandable given how rare the tissue is and does not lessen the tremendous value of this resource. Fellow researchers can now confirm and build upon the knowledge that Rayon and colleagues have laid out.

No doubt this resource will have a great impact and become a reference for the community.

Comments for the author

Classification of central nervous cells
- "Nevertheless, attempts to use standard unsupervised methods to cluster progenitors were unsuccessful at classifying cells into the known dorsoventral domains. Similar approaches with neurons were also unsuccessful. This suggested that, similar to mouse, the complexity and combinatorial expression profile of genes in both progenitors and neurons preclude the use of unsupervised approaches.". This is surprising and intriguing given the beautiful separation of the different neural progenitor domains when looking at proteins of well-known marker genes. Because getting intact cells from the complex developing human spinal cord may be challenging, do the authors think that ambient RNA from broken or dead cells is adding noise and making unsupervised clustering approaches challenging? Thinking of other potential technical limitations, have the authors checked if the sequencing depth per cell is enough to obtain a representative cell transcriptome? For example, does the number of genes plateau before or around 10k reads per cell? Any insights on these may be useful for future attempts at expanding the single-cell atlas of the developing spinal cord.

Dynamics of neurogenesis in the human spinal cord
- "More detailed inspection indicated that at CS12, the proportion of p3 and pMN ventral progenitors compared to pI and pD was higher in human than mouse (FIG 6F). The increased proportion of ventral progenitors persisted at CS14, but by CS17 the size of the ventral domains was comparable between mouse and human (FIG 6F)". What is the percentage difference between the human and mouse values? The human data is quite limited (only one embryo, two technical replicates) and the differences don't seem big. I suggest adding the percentage values into the text (so that readers can more clearly see the differences) and maybe a sentence mentioning that this is what the limited data suggests. If possible, it will also be good to confirm this by immunofluorescence. The human samples available for this will probably also be limited but can support/confirm the finding.

© 2021. Published by The Company of Biologists under the terms of the Creative Commons Attribution License (https://creativecommons.org/licenses/by/4.0/).

- "There was a reduction in the proportion of progenitors in the pD domains from CS14 to CS17, consistent with a slower rate of dorsal neurogenesis in human indicative of a delay in dorsal neurogenesis (FIG 6F).".
There seems to be something wrong with this sentence. I guess the authors meant "[...]from CS17 to CS14 consistent with a slower rate of dorsal neurogenesis in human" or ""[...]from CS17 to CS14, indicative of a delay in dorsal neurogenesis". In any case, do the authors think this that decrease is significant? Again they are limited by having data from only from one embryo for each stage and the range around the mean percentage per sample is quite broad. I am not convinced that the data supports their claim.

- "Together, the analysis indicated that, although mouse and human display an overall similar pattern of neurogenesis, the human neural tube has a higher initial proportion of pMN and p3 progenitors and these undergo a higher relative rate of neurogenesis. By contrast, dorsal neurogenesis persists for a longer period in human than mouse." How do the authors conclude the rate of neurogenesis from the relative proportion of neural progenitors? To infer the rate of neurogenesis (i.e. how often neurons are generated), don't they need to bring into the equation the number of neurons being generated? Maybe each data point in Fig 6F should be the ratio of progenitors to neurons (the ratio between Fig 6 A and B). How many neurons each progenitor is generating may not be the same in mouse and human (due for example to the longer human cell cycle length) so looking at the progenitor-to-neuron ratio would be more appropriate.

Minor comments:
- Fig. 3B If the authors could make the plot in panel B a touch wider, it will be easier to read the gene names.
- Fig. 3D Please specify in the figure legend how many embryos were analysed.
- "In addition, we identified a small number of cells (n=77), present from CS12 onwards, expressing SOX10 SOX9, PDGFRA and S100, characteristic of oligodendrocytes (FIG6G).". There is a B missing in S100.
- Fig. 6 figure legend: "Vertical bars indicate the range around the mean of proportions per replicate.". Do the authors mean per sample?
- Fig 7A: The black arrows are difficult to see, please make bigger (or add a big arrow as in Fig S6). The colour legends should also be enlarged in Fig 7A and Fig S6.
- "Moreover, the analysis highlighted a temporal trajectory within some progenitors including p3, pMN, and p2 in mouse (FIG 7A, FIG S6B)," This is difficult to see in the plot. Maybe the authors can label glia like they labelled neurons or mark the gliogenesis trajectory.
- "To investigate whether Olig2+/Nkx2.2+ coexpressing cells had a distinct transcriptional signature, we compared DP cells." The DP acronym is probably unnecessary. Why not write Olig2+/Nkx2.2+ cells? If the authors decide for DP cells, revise the text for consistency (sometimes double positive cells sometimes DP cells).

<u>Reviewer 2</u>

Advance summary and potential significance to field

This is a resource paper that reports the transcriptome at the single cell level of human developing spinal cord over 4 timepoints. The timepoints are those critical for spinal cord neurogenesis and neuronal subtype specification. The data are high quality, clearly presented, and the comparisons with mouse spinal cord development are carefully and accurately represented. This paper is highly significant for the field --there is much more focus on cortical development—but this paper is the first to report the cell types during human spinal cord development.

The data presented will be a valuable resource for the field. This group also reported one of the data sets for the single cell transcriptome in mouse spinal cord development and the comparison is important in going back and forth between mouse and human models to study normal development and neurodevelopmental disorders.

© 2021. Published by The Company of Biologists under the terms of the Creative Commons Attribution License (https://creativecommons.org/licenses/by/4.0/).

Comments for the author

I have no suggestions for improving the submitted manuscript. It was a pleasure to read. I am looking forward to exploring the processed single-cell sequencing data that will be made available at the cell browser https://shiny.crick.ac.uk/scviewer/neuraltube/.

First revision

Author response to reviewers' comments

Reviewer 1 Advance Summary and Potential Significance to Field:
Rayon and colleagues report the transcriptomic profiling of the cells that make up the developing human neural tube from gestational week 4 to 7 (CS 12, CS14, CS17, and CS19). A lot is known about spinal cord development in other vertebrates, but we know surprisingly little about human spinal cord development, mostly due to the difficulty to access tissue from human embryos. This is the first single-cell atlas of the developing human neural tube to date and will be a tremendous resource for the community.

Rayon and colleagues use single-cell RNA-sequencing to identify progenitor and neuronal cell types of the central and peripheral nervous system of the human neural tube. They go on to compare these cell types with those previously identified in the mouse neural tube at equivalent developmental stages (Delile 2019 Development). Human and mouse cell types seem to share most of their transcriptional signature, however, Rayon and colleagues found intriguing differences too. For example, having transcriptomic insight into OLIG2+/NKX2.2+ cells in the human neural tube allowed the authors to propose that these cells are likely to be oligodendrocyte precursor cells and that oligodendrogenesis may start earlier in humans than in mice. The authors also make use of RNA velocity to infer the developmental trajectories of different neural progenitors as they give rise to neurons, and associated changes in gene expression. This type of analysis can inform strategies for in vitro generation of specific neuronal subtypes and/or help fine-tune existing protocols.

Besides providing the most detailed insight into human spinal cord cells, Rayon and colleagues have done a great job at making the data and the code accessible to everybody. Their single cell portal (https://shiny.crick.ac.uk/scviewer/neuraltube/) works beautifully and will become a reference for the community.

The only limitation of the paper is only having cells from one embryo for each stage. Most of my comments below relate to this, as I think that the authors should keep this in mind when making some conclusions. The small sample size is more than understandable given how rare the tissue is and does not lessen the tremendous value of this resource. Fellow researchers can now confirm and build upon the knowledge that Rayon and colleagues have laid out.

No doubt this resource will have a great impact and become a reference for the community.

Reviewer 1 Comments for the Author:
Classification of central nervous cells
- "Nevertheless, attempts to use standard unsupervised methods to cluster progenitors were unsuccessful at classifying cells into the known dorsoventral domains. Similar approaches with neurons were also unsuccessful. This suggested that, similar to mouse, the complexity and combinatorial expression profile of genes in both progenitors and neurons preclude the use of unsupervised approaches.". This is surprising and intriguing given the beautiful separation of the different neural progenitor domains when looking at proteins of well-known marker genes. Because getting intact cells from the complex developing human spinal cord may be challenging, do the authors think that ambient RNA from broken or dead cells is adding noise and making unsupervised clustering approaches challenging? Thinking of other potential technical limitations, have the authors checked if the sequencing depth per cell is enough to obtain a representative

© 2021. Published by The Company of Biologists under the terms of the Creative Commons Attribution License (https://creativecommons.org/licenses/by/4.0/).

cell transcriptome? For example, does the number of genes plateau before or around 10k reads per cell? Any insights on these may be useful for future attempts at expanding the single-cell atlas of the developing spinal cord.

The reviewer raises a good point, as we could have used the expression of well-known markers (Fig 1E) to manually classify the clusters in the UMAP. Manual cluster-by-cluster annotation is frequently used to classify similar datasets, but we tried to classify our dataset (i) unbiasedly using scCATCH and CellAssign and (ii) using a knowledge matrix to directly compare the human data with the mouse scRNAseq previously published. This approach avoids subjective cluster-by-cluster classification, as it is insensitive to cluster resolution.

Ambient RNA from broken and dead cells could add noise to the dataset, but we do not think this is the source of failure of identification of cell types using unsupervised clustering methods. We think that our inability to unbiasedly classify the cells is due to the fact that the algorithms are developed to identify cell types in adult tissues with markedly distinct transcriptomes that result in discrete cell clusters in scRNAseq analyses. These approaches failed to classify the subtly different cell types with similar expression profiles that change across timepoints. We note that the standard algorithms performed similarly on our mouse neural tube scRNAseq dataset as they did on human.

We have applied both scCATCH v2.1 and CellAssign to unbiasedly classify cells in our dataset. scCATCH annotates cell types of clusters from scRNAseq data using a reference database that contains 353 cell types and related 686 subtypes associated with 184 tissue types reproducibly without prior knowledge (Shao et al., 2020). Cell type classification using such approaches are therefore limited to the level of granularity described by the database. scCATCH retrieved broad classes of cell types (Neuron Progenitors, progenitor cell, precursor cell, astrocytes, …) but lacked the detailed classification of the developing spinal cord that we could predict using previous knowledge on the developing neural tube. We employed CellAssign (Zhang et al., 2019) using our knowledge matrix to annotate cell types, and this gave the predicted cell types and probabilities for each cell type. However, CellAssign failed to annotate a large proportion of the sequenced cells.

Regarding other potential technical limitations, the samples contain sufficient sequencing depth per cell and passed standard 10X Cell Ranger QC tests. Of note, low-quality cells with low UMIs per cells (limited complexity) identified by Cell Ranger were removed from the analysis (Low quality - Fig S1H). In addition, the quality of the human dataset was comparable to the mouse dataset.

Dynamics of neurogenesis in the human spinal cord
- "More detailed inspection indicated that at CS12, the proportion of p3 and pMN ventral progenitors compared to pI and pD was higher in human than mouse (FIG 6F). The increased proportion of ventral progenitors persisted at CS14, but by CS17 the size of the ventral domains was comparable between mouse and human (FIG 6F)".
What is the percentage difference between the human and mouse values? The human data is quite limited (only one embryo, two technical replicates) and the differences don't seem big. I suggest adding the percentage values into the text (so that readers can more clearly see the differences) and maybe a sentence mentioning that this is what the limited data suggests. If possible, it will also be good to confirm this by immunofluorescence. The human samples available for this will probably also be limited but can support/confirm the finding.

We agree with the reviewer that the human data is limited. We aimed to describe the data as accurately as possible and have now included the percentage values in the main text and a supplementary table (Table S5) with the proportions of cells per domain and timepoint. In addition, we have now performed immunofluorescence of PAX3 (pD), OLIG2 (pMN), NKX2.2 (p3) and ARX (FP) in mouse E9.5 and E11.5 embryos (n=3 per stage, 1 section each) and human CS12, CS17 embryos (n=1 per stage, 3 sections). The stainings confirm the differences observed in the domain sizes between human and mouse at early stages (see new Fig 6 and S6).
The pD and pMN size differences in human compared to mouse are no longer evident by CS17 (Fig S6), and a clear reduction of the ventral domain size by CS17 is observed by immunofluorescence staining on mouse and human samples (New Fig S6).

© 2021. Published by The Company of Biologists under the terms of the Creative Commons Attribution License (https://creativecommons.org/licenses/by/4.0/).

We have edited the text accordingly "More detailed inspection indicated that in the CS12 embryo, the proportion of p3 and pMN ventral progenitors compared to pI and pD was higher in human than mouse (pD and pI human 55% cells, mouse 72% cells; pMN and p3 human 22% cells, mouse 16% cells) (FIG 6, S6). The increased proportion of ventral progenitors persisted in the CS14 embryo (pMN and p3 human 11% cells, mouse 5% cells), but in the CS17 single embryo the size of the ventral domains was comparable between mouse and human (pMN and p3 in human and mouse 3%) (FIG 6F, S6)"

- "There was a reduction in the proportion of progenitors in the pD domains from CS14 to CS17, consistent with a slower rate of dorsal neurogenesis in human indicative of a delay in dorsal neurogenesis (FIG 6F).". There seems to be something wrong with this sentence. I guess the authors meant "[...]from CS17 to CS14, consistent with a slower rate of dorsal neurogenesis in human" or ""[...] from CS17 to CS14, indicative of a delay in dorsal neurogenesis". In any case, do the authors think this that decrease is significant? Again, they are limited by having data from only from one embryo for each stage and the range around the mean percentage per sample is quite broad. I am not convinced that the data supports their claim.

As the reviewer points out, this conclusion is limited by the little number of samples we analysed. We have now included the cell percentages, confirmed pD and pMN sizes by immunofluorescence and examined rates of neurogenesis (see comment below).

- "Together, the analysis indicated that, although mouse and human display an overall similar pattern of neurogenesis, the human neural tube has a higher initial proportion of pMN and p3 progenitors and these undergo a higher relative rate of neurogenesis. By contrast, dorsal neurogenesis persists for a longer period in human than mouse." How do the authors conclude the rate of neurogenesis from the relative proportion of neural progenitors? To infer the rate of neurogenesis (i.e. how often neurons are generated), don't they need to bring into the equation the number of neurons being generated? Maybe each data point in Fig 6F should be the ratio of progenitors to neurons (the ratio between Fig 6 A and B). How many neurons each progenitor is generating may not be the same in mouse and human (due for example to the longer human cell cycle length) so looking at the progenitor-to-neuron ratio would be more appropriate.

As the reviewer points out, single cell analysis does not provide direct information about rate of neurogenesis, and it does not consider cell cycle length. Even though the ratio of neurons over progenitors in a single time point does not accurately provide the rate of neurogenesis, as the neurons at a particular timepoint come from progenitors of an earlier stage, it is a reasonable proxy to infer rates of neurogenesis. As suggested by the reviewer, we have now compared the ratio of neurons over progenitors in dorsal and ventral domains per timepoint in mouse and human:

© 2021. Published by The Company of Biologists under the terms of the Creative Commons Attribution License (https://creativecommons.org/licenses/by/4.0/).

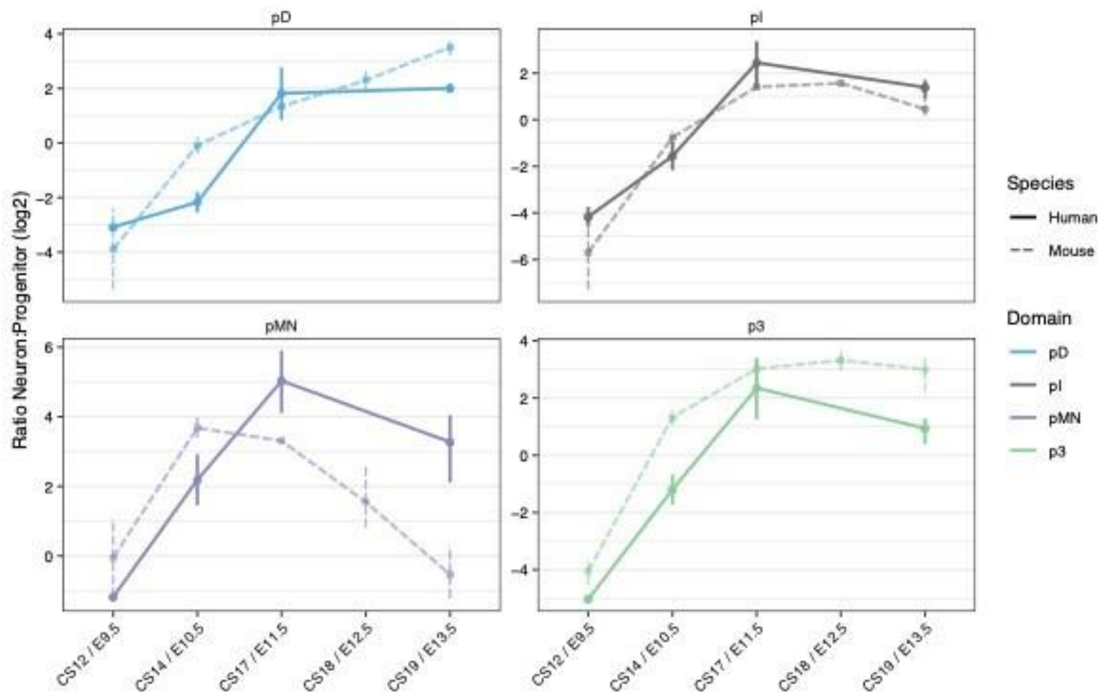

We can observe that there are few neurons born at early time points (CS12, CS14), and that the neuronal output is increased in all domains from CS17 onwards. Neurogenesis starts earlier in the pMN in mouse and human (from CS14) than in the rest of the domains, as expected. We find that the rate of MN differentiation in human persists at a higher rate in human from CS17. By contrast, the rate of neurogenesis is relatively higher in the mouse p3 than in the human. Given the higher proportion of pMN cells in human (see previous comment above about domain size and cell proportions), and that these undergo a higher relative rate of neurogenesis, we have modified the manuscript to read, “together, the analysis indicated that, although mouse and human display an overall similar pattern of neurogenesis, the human neural tube has a higher initial proportion of pMN and p3 progenitors and pMN cells undergo a higher relative rate of neurogenesis.”

The rate of neurogenesis in the pD and pI domains is comparable between mouse and human across timepoints. However, neurogenesis in the pD domain in the CS17 and CS19 embryos appears to be constant, whereas there is a marked increase in pD progenitors from E11.5 to E13.5 in mouse. We interpreted this as an indication of delayed neurogenesis in dorsal progenitors, but the referee is right to point out that the data may not be enough to interpret the duration of neurogenesis in human. Thus, we have toned down the statement “By contrast, dorsal neurogenesis may persist for a longer period in human than mouse.”

Altogether, this section in the manuscript has been updated to read, “Next, we compared the ratio of neurons over progenitors across domains per timepoints as a proxy of the rate of neurogenesis. Whereas there was little neurogenesis between CS12 and CS14, the neuronal output increased in all domains from CS17 onwards (Fig S6A). As expected, there was an earlier onset of neurogenesis in the mouse and human pMN than in the rest of the domains (Fig S6A), but the rate of MN differentiation in human persists at a higher rate from CS17 compared to mouse. In contrast, the rate of neurogenesis in the pD and pI domains is comparable between mouse and human across timepoints (Fig S6). Neurogenesis in the pD domain in the CS17 and CS19 embryos appeared to be constant, whereas there was a marked increase in pD progenitors from E11.5 to E13.5 in mouse (FIG S6), consistent with a slower rate of dorsal neurogenesis in human. Together, the analysis indicated that, although mouse and human display an overall similar pattern of neurogenesis, the human neural tube has a higher initial proportion of pMN and p3 progenitors and pMN cells undergo a higher relative rate of neurogenesis. By contrast, dorsal neurogenesis may persist for a longer period in human than mouse. Further validation including more embryos spanning later timepoints will be needed to determine if human neurogenesis is delayed.”

© 2021. Published by The Company of Biologists under the terms of the Creative Commons Attribution License (https://creativecommons.org/licenses/by/4.0/).

Minor comments:
- Fig. 3B If the authors could make the plot in panel B a touch wider, it will be easier to read the gene names.

We have increased the plot in 3B for clarity. The genes are the same as in 3A and 3C.

- Fig. 3D Please specify in the figure legend how many embryos were analysed.
We have included the number of embryos and sections in the figure legends and in the methods section.

- "In addition, we identified a small number of cells (n=77), present from CS12 onwards, expressing SOX10, SOX9, PDGFRA and S100, characteristic of oligodendrocytes (FIG6G).". There is a B missing in S100.

Thank you. Corrected.

- Fig. 6 figure legend: "Vertical bars indicate the range around the mean of proportions per replicate.". Do the authors mean per sample?

Yes. We have corrected the figure legend accordingly.

- Fig 7A: The black arrows are difficult to see, please make bigger (or add a big arrow as in Fig S6). The colour legends should also be enlarged in Fig 7A and Fig S6.

We have now increased the arrows and legends as suggested for clarity in Fig 7A and Fig S7B.

- "Moreover, the analysis highlighted a temporal trajectory within some progenitors including p3, pMN, and p2 in mouse (FIG 7A, FIG S6B)," This is difficult to see in the plot. Maybe the authors can label glia like they labelled neurons or mark the gliogenesis trajectory.

The temporal trajectory of progenitors (gliogenesis) is now indicated with an orange arrow in pMN (FIG 7A), p3 and p2 progenitors, and the figure legend has been amended accordingly.

- "To investigate whether Olig2+/Nkx2.2+ coexpressing cells had a distinct transcriptional signature, we compared DP cells." The DP acronym is probably unnecessary. Why not write Olig2+/Nkx2.2+ cells? If the authors decide for DP cells, revise the text for consistency (sometimes double positive cells, sometimes DP cells).

For clarity and consistency, we have now edited the text and changed all reference to DP cells for Olig2+/Nkx2.2+ cells. For lack of a better annotation system, we refer to *Olig2+/Nkx2.2+* cells for mouse, *OLIG2+/NKX2.2+* in human, and Olig2+/Nkx2.2+ when we refer to cells in the two species.

Reviewer 2 Advance Summary and Potential Significance to Field:
This is a resource paper that reports the transcriptome at the single cell level of human developing spinal cord over 4 timepoints. The timepoints are those critical for spinal cord neurogenesis and neuronal subtype specification. The data are high quality, clearly presented, and the comparisons with mouse spinal cord development are carefully and accurately represented. This paper is highly significant for the field --there is much more focus on cortical development— but this paper is the first to report the cell types during human spinal cord development.

The data presented will be a valuable resource for the field. This group also reported one of the data sets for the single cell transcriptome in mouse spinal cord development and the comparison is important in going back and forth between mouse and human models to study normal development and neurodevelopmental disorders.

Reviewer 2 Comments for the Author:
I have no suggestions for improving the submitted manuscript. It was a pleasure to read. I am looking forward to exploring the processed single-cell sequencing data that will be made available at the cell browser https://shiny.crick.ac.uk/scviewer/neuraltube/.

© 2021. Published by The Company of Biologists under the terms of the Creative Commons Attribution License (https://creativecommons.org/licenses/by/4.0/).

We thank the reviewer for their positive feedback.

REFERENCES

Shao, X., Liao, J., Lu, X., Xue, R., Ai, N. and Fan, X. (2020). scCATCH: Automatic Annotation on Cell Types of Clusters from Single-Cell RNA Sequencing Data. *iScience* **23**, 100882.
Zhang, A. W., O'Flanagan, C., Chavez, E. A., Lim, J. L. P., Ceglia, N., McPherson, A., Wiens, M., Walters, P., Chan, T., Hewitson, B., et al. (2019). Probabilistic cell-type assignment of single-cell RNA-seq for tumor microenvironment profiling. *Nature Methods* **16**, 1007–1015.

Second decision letter

MS ID#: DEVELOP/2021/199711

MS TITLE: Single cell transcriptome profiling of the human developing spinal cord reveals a conserved genetic programme with human specific features

AUTHORS: Teresa Rayon, Rory J Maizels, Christopher Barrington, and James Briscoe
ARTICLE TYPE: Techniques and Resources Article

I am happy to tell you that your manuscript has been accepted for publication in Development, pending our standard ethics checks.

Reviewer 1

Advance summary and potential significance to field

(The summary I wrote about the author's initial submission still describes my thoughts on the advance made in the revised paper and its potential significance to the field)
Rayon and colleagues report the transcriptomic profiling of the cells that make up the developing human neural tube from gestational week 4 to 7 (CS12, CS14 CS17, and CS19). A lot is known about spinal cord development in other vertebrates, but we know surprisingly little about human spinal cord development, mostly due to the difficulty to access tissue from human embryos.
This is the first single-cell atlas of the developing human neural tube to date and will be a tremendous resource for the community.
Rayon and colleagues use single-cell RNA-sequencing to identify progenitor and neuronal cell types of the central and peripheral nervous system of the human neural tube. They go on to compare these cell types with those previously identified in the mouse neural tube at equivalent developmental stages (Delile 2019 Development). Human and mouse cell types seem to share most of their transcriptional signature, however, Rayon and colleagues found intriguing differences too. For example, having transcriptomic insight into OLIG2+/NKX2.2+ cells in the human neural tube allowed the authors to propose that these cells are likely to be oligodendrocyte precursor cells and that oligodendrogenesis may start earlier in humans than in mice. The authors also make use of RNA velocity to infer the developmental trajectories of different neural progenitors as they give rise to neurons, and associated changes in gene expression. This type of analysis can inform strategies for in vitro generation of specific neuronal subtypes and/or help fine-tune existing protocols.
Besides providing the most detailed insight into human spinal cord cells, Rayon and colleagues have done a great job at making the data and the code accessible to everybody. Their single cell portal (https://shiny.crick.ac.uk/scviewer/neuraltube/) works beautifully and will become a reference for the community.

© 2021. Published by The Company of Biologists under the terms of the Creative Commons Attribution License (https://creativecommons.org/licenses/by/4.0/).

Comments for the author

The authors addressed all my comments in the revised version of the paper. I look forward to seeing their beautiful work published in Development, and to keep exploring their data!

Reviewer 2

Advance summary and potential significance to field

This is a resource paper that reports the transcriptome at the single cell level of human developing spinal cord over 4 timepoints. The timepoints are those critical for spinal cord neurogenesis and neuronal subtype specification. The data are high quality, clearly presented, and the comparisons with mouse spinal cord development are carefully and accurately represented. This paper is highly significant for the field --there is much more focus on cortical development—but this paper is the first to report the cell types during human spinal cord development.

Comments for the author

Responses to reviewer comments and changes made to the manuscript are clear--this is an excellent study.

© 2021. Published by The Company of Biologists under the terms of the Creative Commons Attribution License (https://creativecommons.org/licenses/by/4.0/).
